# Role of serotonergic dorsal raphe neurons in hypercapnia-induced arousals

**DOI:** 10.1038/s41467-020-16518-9

**Published:** 2020-06-02

**Authors:** Satvinder Kaur, Roberto De Luca, Mudasir A. Khanday, Sathyajit S. Bandaru, Renner C. Thomas, Rebecca Y. Broadhurst, Anne Venner, William D. Todd, Patrick M. Fuller, Elda Arrigoni, Clifford B. Saper

**Affiliations:** Department of Neurology, Division of Sleep Medicine, and Program in Neuroscience, Beth Israel Deaconess Medical Center and Harvard Medical School, Boston, MA 02215 USA

**Keywords:** Wakefulness, Neural circuits

## Abstract

During obstructive sleep apnea, elevation of CO_2_ during apneas contributes to awakening and restoring airway patency. We previously found that glutamatergic neurons in the external lateral parabrachial nucleus (PBel) containing calcitonin gene related peptide (PBel^CGRP^ neurons) are critical for causing arousal during hypercapnia. However, others found that genetic deletion of serotonin (5HT) neurons in the brainstem also prevented arousal from hypercapnia. To examine interactions between the two systems, we showed that dorsal raphe (DR) 5HT neurons selectively targeted the PBel. Either genetically directed deletion or acute optogenetic silencing of DR^Sert^ neurons dramatically increased the latency of mice to arouse during hypercapnia, as did silencing DR^Sert^ terminals in the PBel. This effect was mediated by 5HT_2a_ receptors which are expressed by PBel^CGRP^ neurons. Our results indicate that the serotonergic input from the DR to the PBel via 5HT_2a_ receptors is critical for modulating the sensitivity of the PBel^CGRP^ neurons that cause arousal to rising levels of blood CO_2_.

## Abstract (web summary)

Dorsal raphe 5HT(DR^Sert^) neurons regulate arousal from hypercapnia by their projections to the neurons in the external lateral parabrachial nucleus (PBel) that are glutamatergic and also express calcitonin gene related peptide (PBel^CGRP^). The DR^Sert^ input to the PBel modulates the arousal system to rising levels of blood CO_2_, and may be mediated by 5HT_2a_ receptors on the PBel^CGRP^ neurons.

## Introduction

Obstructive sleep apnea is a sleep-related breathing disorder characterized by repeated cycles of upper airway collapse during sleep causing apnea, followed by arousal that re-establishes the airway. Although these arousals are important and critical to re-establishing breathing, they also repeatedly disrupt sleep and prevent entry into deeper states of sleep^1,2^. The resulting sleep fragmentation leads to daytime sleepiness and increased risk of cardiovascular and metabolic disease^3,4^. Preventing arousal from sleep while augmenting the increase in respiratory drive that reinitiates breathing could potentially ameliorate this outcome^5^, but will require understanding the circuits that mediate both aspects of the response to apnea. Here we study the brain circuit that mediates the arousal to elevated CO_2_, a key sensory stimulus during apnea^6^.

We have recently shown that neurons in the external lateral parabrachial nucleus that express calcitonin gene-related peptide (PBel^CGRP^ neurons) are required for EEG arousal, but not the increase in respiratory drive, induced by hypercapnia. Interestingly, deleting the vesicular glutamate transporter from the PBel causes the same effect as killing the PBel^CGRP^ neurons, so it is likely that the CO_2_ arousal response is mediated by the glutamate, not the CGRP in these neurons. However, the CGRP provides a convenient genetic marker for this cell population. Chemogenetic activation of PBel^CGRP^ neurons caused wakefulness and optogenetically driving them at 10 or 20 Hz produced short latency arousals^7^. We also found that PBel^CGRP^ neurons mediate these arousals chiefly via their inputs to the basal forebrain with inputs to the central nucleus of amygdala and the lateral hypothalamus playing a smaller role^7^.

In addition to the PBel^CGRP^ neurons, Richerson, Buchanan, Dymecki and their colleagues have emphasized the importance of serotonergic neurons for CO_2_ arousal. They point out that many serotonin neurons in the medullary raphe and the dorsal raphe (DR) are CO_2_ (or pH) responsive^8,9^ and that their absence during development in Lmx1b^f/f/p^::ePet1-Cre mice results in frequent apneas and high infant mortality as well as impaired arousal to CO_2_ in adults^10–12^. In these mice CO_2_ responsiveness can be restored by a 5HT_2a_ receptor agonist, suggesting that serotonergic neurons play a modulatory role in the CO_2_ response, but that the CO_2_ sensing by 5HT neurons was not required for CO_2_ arousal^13^. However, the serotonin neurons and their neuronal targets mediating the effect of the serotonin system on CO_2_ arousal have not been identified. In this regard, DR neurons are known to project to the lateral PB^14–16^, are CO_2_ responsive, and injecting the DR with low pH (acidic) solution causes wakefulness in wild-type mice, but not in mice lacking serotonin neurons^17^. Based on the proposed role of the DR serotonergic neurons in CO_2_ chemosensitivity, we hypothesized that the DR may supply a serotonergic input to the PBel^CGRP^ neurons via 5HT_2a_ receptors to sensitize them to CO_2_-responsive inputs. To test this hypothesis, we examined the role of the DR serotonergic neurons in CO_2_ arousal first by deleting them genetically, then by activating them chemogenetically, and finally by inhibiting their cell bodies and then their terminals optogenetically with archaerhodopsin T (ArchT).

## Results

### Selective deletion of DR^Sert^ neurons

**Fig. 1:**
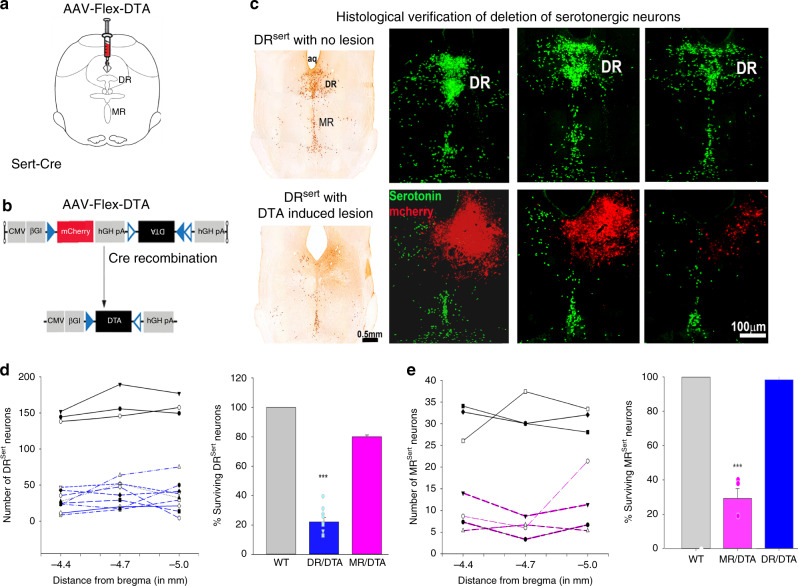
AAV-Flex-DTA induced deletion of DR^Sert^ neurons. The experimental strategy involved (**a**) injection of the viral vector that produced Cre-dependent expression of the diphtheria toxin A subunit (**b**) in the dorsal raphe of Sert-Cre-::L10 mice or wild-type littermates (WT). Injection of the vector deleted nearly all serotonergic neurons at the injection site in the Sert-Cre animals. This is seen in (**c**) the top row showing three levels from rostral-to caudal from an intact animal and the lower row from an animal that received an injection of the vector. Note that at low magnification (left) with brown immunostaining for serotonin, that the DR contains almost no surviving neurons, while the MR is largely intact. At higher magnification (right panels) fluorescence images show the location of the injection site (marked by red stained intact non-serotonergic neurons) and that there are no surviving serotonin neurons within the boundaries of the red injection site. The loss of DR^Sert^ and MR^Sert^ neurons after DR injections sites (**d**), *n* = 9; DR-DTA, neuronal counts from each mouse is represented in left panel of **d**, with average percentage of neuronal survival on the right), where DR-DTA group (*n* = 9); WT (*n* = 3); MR-DTA (*n* = 4) were significantly different (F_1,8_ = 126.6; *P* < 0.001, One-way ANOVA with multiple comparisons). After MR injection sites (**e**), MR-DTA (*n* = 4) is compared with intact WT animals (*n* = 3) and DR-DTA (*n* = 9), and these groups showed significant difference (F_1,5_ = 86.5; *P* < 0.001, one-way ANOVA with multiple comparison). The error bars in **d** and **e** represent the standard error of mean (SEM). ***represents *P* < 0.001, one-way ANOVA to compare the groups in **d** and **e**. Cerebral aqueduct, aq; dorsal raphe, DR; median raphe, MR. Fig. 1a shows a drawing of the mouse brain at the level of the mid-DR (equivalent to plane −4.6 mm from bregma, of the mouse brain atlas)^53^.

We injected AAV-Flex-DTA (Fig. 1a, b) into the DR region of Sert-Cre::L10-GFP mice (which express Cre-recombinase under the serotonin transporter (Sert) promoter, driving Cre-dependent expression of GFP fused with the L10 ribosomal protein, *n* = 13; WT, *n* = 6; Fig. 1c, also see Supplementary Fig. 1 showing validation of the Sert-Cre mice). The extent of deletion of serotonin neurons in the DR was estimated by counting the remaining L10-GFP + cell bodies in the DR (Fig. 1c). In nine mice with well-placed injections in the DR, the number of L10-GFP neurons was reduced by 77.9 ± 2.9% (*F*_1,8_ = 126.6; *P* < 0.001) (Fig. 1d), but serotonergic neurons in the median raphe nucleus (MR^Sert^ cells) were spared (98.4% surviving MR^Sert^ cells; Fig. 1e). In four mice, more ventral injection of the same viral vector (Fig. 1e) resulted in loss of 70.8 ± 5.7% of MR^Sert^ cells (*F*_1,5_ = 86.5; *P* < 0.001), but despite leakage of some virus back along the needle track largely spared the DR^Sert^ cells (80% surviving DR cells; Fig. 1d). Nissl and TH staining of the brain sections from Sert-L10 mice with serotonin neuron deletions demonstrated that the other cell groups in the area remained intact (Supplementary Fig. 2). Wild-type (WT) mice injected with the same AAV (Control-WT) and Sert-Cre::L10-GFP mice injected with an AAV-DIO-ChR2-mCherry vector as controls showed no apparent cell loss.

**Fig. 2:**
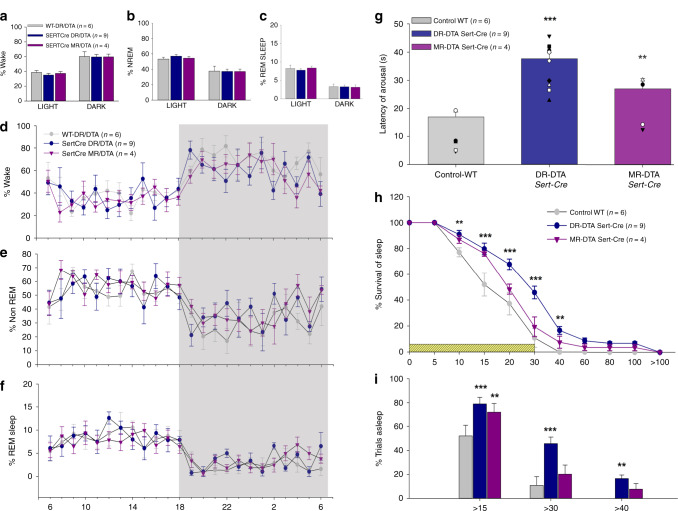
Effect of deletions of serotonergic neurons on spontaneous sleep–wake (a–f) and CO_2_ arousal (g–i). Deletion of serotonin neurons in the DR (Sert-Cre DR-DTA, *n* = 9) or MR (Sert-Cre MR-DTA, *n* = 4) compared with the wild-type (WT, *n* = 6) had no effect on the percentage of time spent (mean ± SEM) in wake (**a**), non-REM sleep (**b**) or REM sleep (**c**) during both the 12 h of light and dark phases. The hour-wise comparison of the wake (**d**) NREM (**e**) and REM sleep (**f**) for the three groups are virtually superimposable. By contrast, during 30 sec exposure to 10% CO_2_, the animals with DR deletions (Sert-Cre DR-DTA, *n* = 9) had a 2.2-fold longer mean latency to arousal and the animals with MR deletions (Sert-Cre MR-DTA, *n* = 4) a 1.6-fold longer latency (**g**). Panel **h** shows the survival curve for the percentage of mice (mean ± SEM) still asleep at various time points after onset of the CO_2_ stimulus. Panel **i** illustrates the percentage of animals (two-way ANOVA for treatments and time, with multiple comparisons to the WT) still asleep at 15 (WT vs. DR-DTA, < *P* = 0.001, vs. MR-DTA, *P* = 0.002), 30 (WT vs. DR-DTA, *P* < 0.001) and 40 sec (WT vs. DR-DTA, *P* = 0.002) after CO_2_ onset. Almost all control animals are awake by 30 sec, but almost 46% of animals with DR^Sert^ deletions and almost 20% with MR^Sert^ deletions failed to arouse by this time point. The error bars in all the graphs (**a**–**i**) represent the SEM. **P < 0.01; ***P < 0.001; based on either one-way (**g**) or two-way ANOVA (**i**, **h**).

The percentages of time spent in wake, NREM, and REM sleep for the 12 h light and dark phases (Fig. 2a–f) in the mice with deletions of either the DR^Sert^ or MR^Sert^ neurons were compared with the control groups with no deletions. We found no significant differences in either the sleep–wake percentages, or in the comparison of the sleep durations and frequency of bouts of wake and NREM and REM sleep, suggesting that DR^Sert^ and MR^Sert^ deletions had no effect on these aspects of sleep architecture.

Control animals showed a mean latency of arousal of 17.0 ± 1.7 sec and arousal within 30 sec of onset of the CO_2_ stimulus almost 90% of the time, whereas animals with DR^Sert^ deletions had a mean latency to arousal of 37.7 ± 2.1 sec (F_2,16_ = 21.3; *P* < 0.001), with no arousal within 30 sec of CO_2_ onset in about 46% of the trials (Fig. 2g). MR deletions also increased the mean latency to arousal to 26.9 ± 3.7 sec, which was significantly (F_2,16_ = 21.3; *P* = 0.022) higher than the WT mice, and produced no arousal within 30 sec in ~20% of the trials (Fig. 2g–i).

**Fig. 3:**
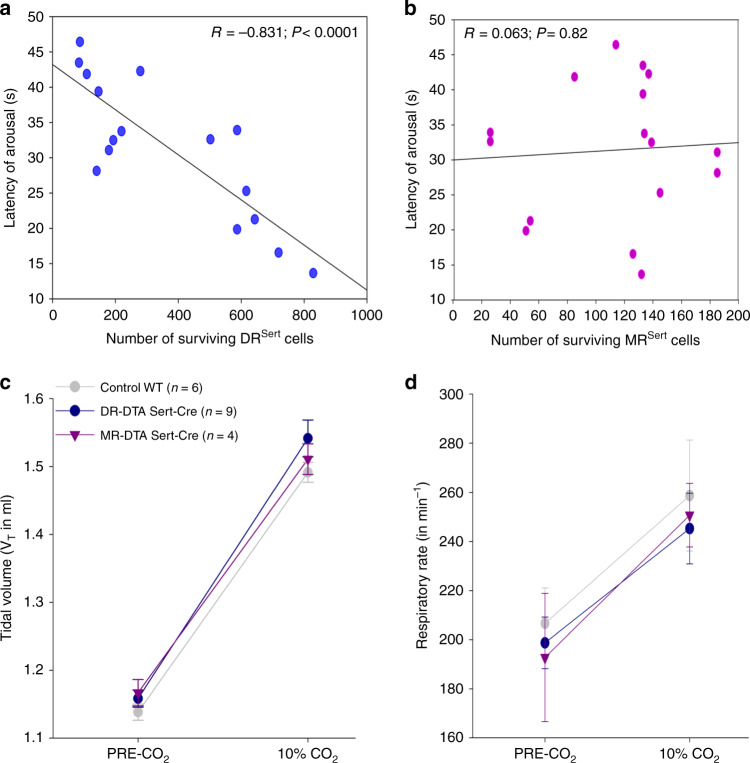
Deletion of DR but not MR serotonin neurons prevents hypercapnic arousal. Correlations of DR^Sert^ and MR^Sert^ neuronal loss to the CO_2_ arousal (**a**–**b**), and effects on the respiration (**c**–**d**). Panels **a** and **b** are the regression graphs showing the correlation of the loss of DR^Sert^ and MR^Sert^ neurons, respectively, to the latency of arousal to CO_2_ across all three treatment groups, WT, DR/DTA, and MR/DTA. Note the close correlation of the loss of DR^Sert^ neurons with mean latency to arousal, even in experiments where the deletion was aimed at the MR (*P* < 0.001, Pearson’s correlation coefficient), and the lack of correlation of loss of MR^Sert^ neurons with latency to CO_2_ arousal (*P* = 0.82). The tidal volume (**c**) and the respiratory rate (**d**) both before and after exposure (mean ± SEM) to CO_2_ did not differ among the treatment groups, indicating that the DR^Sert^ neurons appear to have little role in modulating the ventilatory response to CO_2_. Data in **c** and **d** are presented as mean values ± SEM.

To assess if the increase in latency of arousal correlated with the deletions of serotonergic populations of either the DR or MR, Pearson correlation coefficients were calculated for the numbers of serotonergic cells in the DR and MR compared with the latency of arousal in the three treatment groups (Control, DR^Sert^, and MR^Sert^). We found that the number of remaining DR^Sert^ cells showed a significant (*P* = 0.00006) negative correlation (*r* = −0.83) with latency to arousal (Fig. 3a), whereas the correlation of remaining MR^Sert^ neurons (Fig. 3b) with latency to arousal was not statistically significant (*P* = 0.82).

To assess if the lesions of serotonergic neurons in the DR or MR affected the ventilatory response to CO_2_, we also analyzed the tidal volume (Fig. 3c) and respiratory rate (Fig. 3d) for five breaths before the onset of the CO_2_ stimulus (Pre-CO_2_) and for five breaths just before the mean time to arousal in the control group, in trials where the animal slept for at least 17 sec after onset of CO_2_, in each of the three treatment groups. Neither the tidal volume nor the respiratory rates were statistically different between the three treatment groups, indicating that the lesions had no effect on the ventilatory response to CO_2_.

### Chemogenetic activation of DR^Sert^ neurons

We next injected AAV-DIO-hM3dq-mCherry into the DR of Sert-Cre mice, and verified the expression of mCherry (signifying hM3Dq expressing neurons) in the serotonin immuno-labeled DR^Sert^ neurons^7,18^, although fewer than half of the DR^Sert^ neurons were transfected in individual experiments (Supplementary Fig. 3a and b). Injections of the ligand for hM3Dq, clozapine-N-oxide (CNO) had no significant effect on the percentage of the time spent in either sleep (CNO- 56.31 ± 3.8%; Saline- 53.2 ± 2.6%) or wake (CNO- 43.7 ± 3.8%; Saline- 46.8 ± 2.6%) states (Supplementary Fig. 3d and e), in animals sleeping in the plethysmograph. We then compared the latencies of CO_2_ arousal after injections of either 0.9% saline or CNO (0.3 mg/kg). Activation of the DR^Sert^ neurons by CNO did not change the latency of arousal to CO_2_ (15.8 ± 0.7 sec) when compared with that after injection of saline (16.1 ± 1.3 sec) (Supplementary Fig. 3c). This would suggest that, whereas the DR^Sert^ neurons are necessary for CO_2_ arousal, their firing does not directly cause arousal in the absence of CO_2_, and that when sufficient serotonin is provided, there is a ceiling effect (i.e., additional serotonin release does not further increase CO_2_ response). None of the MR^Sert^ neurons expressed hM3Dq in these experiments.

### Optogenetic inhibition of the DR^Sert^ neurons

**Fig. 4:**
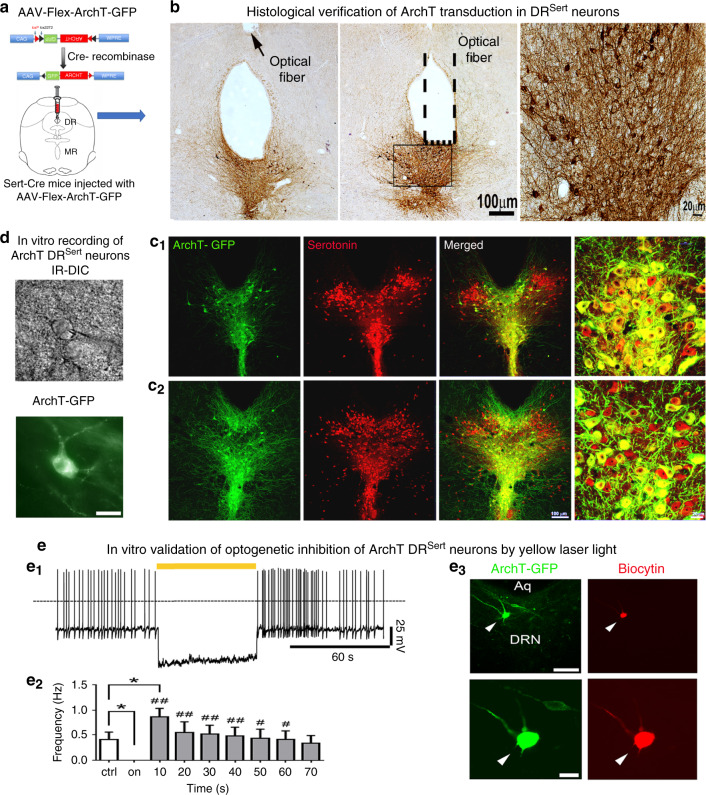
Optogenetic silencing of the DR^Sert^ neurons. The experimental strategy for acute silencing of the DR^Sert^ neurons (**a**) involved expressing the inhibitory opsin ArchT selectively in the DR^Sert^ neurons, by injecting AAV-Flex-ArchT-GFP in the DR of Sert-Cre mice. Photomicrographs in **b** show the histological validation of ArchT transduction (*n* = 6) in the DR^Sert^ neurons at two rostral-to-caudal levels, and their relationship with the tip of the optical fiber targeting the ArchT-DR^Sert^ cells (the flat fiber tip rested on the floor of the cerebral aqueduct; its position is marked by a dotted line and an arrow). To validate that ArchT is expressed only in the serotonin cells (*n* = 6), **c1** and **c2** show photomicrographs from a brain section labeled for both GFP (ArchT- green) and serotonin (red); and merged (double labeling is yellow) showing that nearly all the ArchT-expressing cells were serotoninergic. The fourth panel in both **c1** and **c2** are the same sections represented at higher magnification. Panel **a** shows a drawing of the mouse brain at the level of the mid-DR (equivalent to plane −4.6 mm from bregma, of the mouse brain atlas)^53^. **d**–**e** In vitro brain slice recordings from a DRN ArchT-GFP neuron (**d**) photographed under IR-DIC (top) and under fluorescence optics (bottom; scale bar: 25 µm). **e1** Exposure to 593 nm light (60 s duration) hyperpolarized and silenced action potential firing of DR^Sert^ neurons expressing ArchT. **e2** Bar histogram graph representing the mean firing frequencies before (crtl), during (on) and after (10–70 s; 10 s bins) photoinhibition. One-way ANOVA, *F* = 7.11; *p* < 0.0001 *n* = 6; Holm–Sidak’s multiple comparisons post hoc test; *P = 0.038 ctrl vs light-on; *P = 0.014, ctrl vs 10 s Light-OFF; ^##^*P* < 0.01 and ^#^*p* < 0.05 Light-ON vs Light-OFF. Data in e2 are presented as mean values ± SEM. **e3** Post hoc intracellular biocytin labeling (red with streptavidin-AF-555 in right top panel) of a recorded DRN neuron that expresses ArchT-GFP (green), at low magnification (top left panel; scale bar: 100 µm) and at higher magnification (bottom; scale bar: 20 µm). The results shown in d and e3 were reproduced in *n* = 6 cells that were recorded from two mice.

We have previously reported that optogenetic inhibition of PBel^CGRP^ neuronal cell bodies or their terminals by ArchT can greatly reduce CO_2_ arousal^7^. To determine the mechanism by which the DR^Sert^ neurons promote CO_2_ arousal, we therefore injected AAV-Flex-ArchT-GFP into the DR region in Sert-Cre mice (Fig. 4a–c). The injections transduced 70–80% of DR^Sert^ neurons and nearly all ArchT/GFP positive neurons were serotonin positive (Fig. 4c1 and c2).

Next we tested the responses of DR^Sert^ neurons that express ArchT (Fig. 4d and e) to yellow light by patch clamp recordings in brainstem slices. A 60 sec light exposure hyperpolarized DR^Sert^ neurons that express ArchT (−24.8 ± 10.2 mV, delta from pre-stimulation; *n* = 5) and silenced neuronal firing (*n* = 6; Fig. 4e1). When the yellow light was turned off the membrane potential returned back to values similar to control (membrane potential: −45.6 ± 4.4 mV; *n* = 5), and there was a rebound in firing rate which was greater in the first 10 s after Light-OFF but returned to baseline within 60 sec post light exposure (Fig. 4e2).

**Fig. 5:**
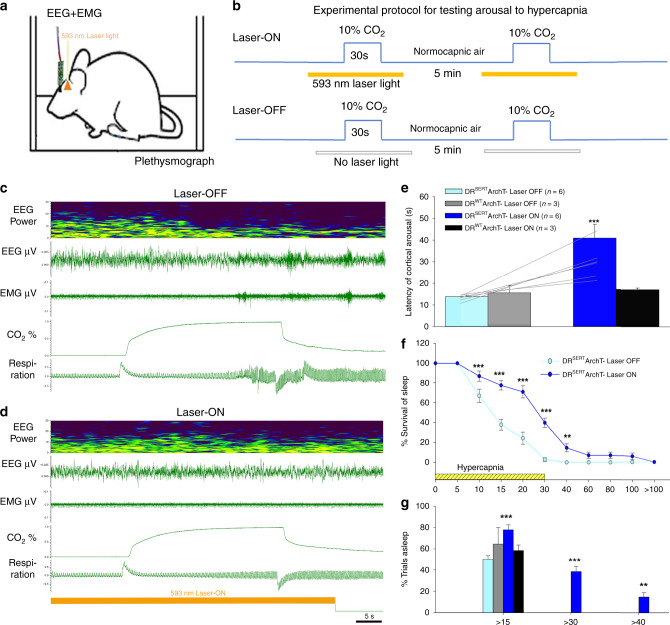
Optogenetic silencing of the DR^Sert^ neurons acutely prevents hypercapnia-induced arousal. Schematic of a mouse (**a**) implanted with EEG/EMG and glass fiber targeting the DR in the experimental protocol to test CO_2_ arousal, as shown in **b**. The 593 nm laser is on for 20 sec before and 10 sec after each 30 sec period of CO_2_ stimulation during the Laser-ON condition. Panels **c**, **d** show the EEG power spectrum, EEG, EMG, CO_2_ level in the chamber, and respiration during representative trials of CO_2_ exposure in Laser-OFF (**c**) and Laser-ON (**d**) conditions. The mouse awoke in 17 sec in response to the rising CO_2_ during Laser-OFF in **c**, as demonstrated by desynchronization of the EEG (decrease of power in lower frequencies; shown in top two traces), increase in EMG (third trace) and sudden increase in the rate and volume of ventilation at the time of EEG arousal (lower trace) in **c**. With Laser-ON (**d**), the mouse did not wake up to the CO_2_ stimulus as can be seen by the presence of synchronized EEG, and minimal EMG activity, during the steadily increasing respiration in response to CO_2_ (there are brief artifacts in the plethysmograph air flow trace at the shift in gas mixtures). Panels **e**–**g** show graphically the effect of acute silencing of the DR^Sert^ neurons on the latency of CO_2_ arousal (mean ± SEM) (**e**) in Laser-OFF and Laser-ON conditions (DR^Sert^ ArchT, *n* = 6; DR^WT^ ArchT, *n* = 3), and the mice in the DR^Sert^ group are shown using line graphs in both Laser conditions. The survival curves (**f**) show the reduction in arousal (mean ± SEM) across the CO_2_ exposure. Panel **g** shows that ~40% of the mice with silencing of DR^Sert^ neurons did not awaken within 30 sec (F_3,14_ = 49.41, *P* < 0.001) of onset of CO_2_ exposure, whereas without the laser the same mice woke up in every trial, and these were statistically compared using the one-way ANOVA followed by multiple comparisons for treatment groups. Data in **e**–**g** are presented as mean values ± SEM. **P < 0.01; ***P < 0.001; one-way (**e**, **g**) or two-way ANOVA (**f**).

In six (of 10) mice injected with AAV-FLEX-ArchT-GFP, we confirmed ArchT expression in the DR^Sert^ neurons and that the tips of the glass fibers were placed above the ArchT-expressing cells. When these six mice were tested for arousal to hypercapnia (Fig. 5b) in the Laser-OFF condition, they showed an arousal latency of 13.8 ± 0.7 sec (Fig. 5c and e), and awakened within 30 sec of CO_2_ onset with every trial (Fig. 5f and g). When tested in the Laser-ON condition, the arousal latency of the mice was tripled (40.9 ± 6.4 sec; F_3,17_ = 11.5; *P* < 0.001; Fig. 5d and e) and in almost 40% of the trials the mice did not wake up within the 30 sec of the CO_2_ stimulus (Fig. 5f and g). The wild-type mice injected with AAV-FLEX-ArchT-GFP showed no ArchT expression and their mean arousal latency with and without the laser remained unchanged (Laser-OFF- 16.8 ± 3.4 sec; Laser-ON- 18.2 ± 0.7 sec), and was similar to the ArchT-expressing group with the Laser-OFF. In no case did the expression of ArchT extend to the MR^Sert^ neurons.

Similar to in our earlier publications^7,18^, we also tested arousal response to normocapnic air (Supplementary Fig. 4a–c), and found that photoinhibition of DR^Sert^ neurons did not affect the spontaneous arousal latency in these mice; the mean latency to arousal to air-air exchange was ~90 sec and ~10% of animals awakened during the 30 sec gas exposure (i.e., 90% failure of latency to arouse within 30 sec; Supplementary Fig. 4). Similarly, the arousal responses to the acoustic stimuli of different strengths (Supplementary Fig. 4d–g) in the mice with optogenetic inhibition of DR^Sert^ neurons, were also not statistically different.

### Origin of serotonergic afferents to the PB

**Fig. 6:**
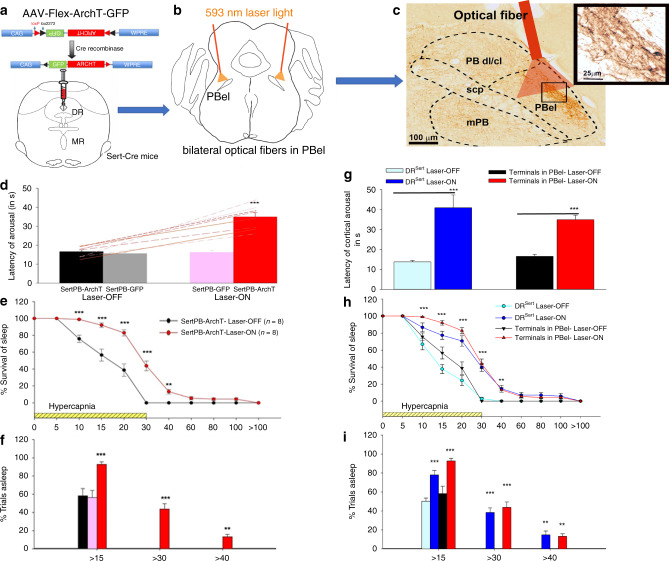
Acute optogenetic silencing of the DR^Sert^ terminals in the PBel blocks hypercapnia-induced arousal. The experimental strategy for acute silencing of the DR^Sert^ terminals in the PBel involved expressing the inhibitory opsin ArchT (injecting AAV-Flex-ArchT-GFP) in Sert-Cre mice to selectively transfect the DR^Sert^ neurons (**a**) and implanting glass fibers bilaterally in PBel (**b**). The photomicrograph in **c** shows a representative brain section illustrating the density and specificity of the ArchT-expressing fibers and terminals in the PBel (immunostained for GFP−brown), and the location of the implanted glass fiber that can direct the laser light (in *n* = 8 mice). Mice were recorded for sleep and breathing in the presence or absence of the laser light (593 nm) during the hypercapnia trials. Acute silencing of the DR^Sert^ terminals in the PBel (SertPB-ArchT, *n* = 8, with the mice represented in the line graph in Laser-OFF and Laser-ON conditions) (**d**) dramatically increased the latency of arousal (mean ± SEM) from 16.6 sec with Laser-OFF to 34.9 sec with the Laser-ON condition. The survival curves (**e**) illustrate the slow arousal to CO_2_ in the Laser-ON condition during which the mice failed to arouse within 30 s during almost 44% of the trials (**f**). **P < 0.01; ***P < 0.001; one-way (**d**, **f**) or two-way ANOVA (**e**). Graphs **g**–**i** compare the data from Fig. 5d–f with Fig. 6d–f on the same graph, to demonstrate that almost the entire effect of silencing the DR^Sert^ cell bodies is achieved just by silencing their terminals in the PBel and these groups (DR^Sert^ and terminals in PB- Laser-ON) were not statistically different (F_1,12_ = 0.98, *P* = 0.34). Data in **d**–**i** are presented as mean values ± SEM. Fig. 6a and b show drawings of the mouse brain at the level of the mid-DR (**a** at −4.6 mm) and mid-PB (**b**), equivalent to plane −5.2 mm from bregma, of the mouse brain atlas)^53^.

Although the PB is known to be innervated diffusely by 5HT axons^19^, the origin of serotonin inputs to its different subnuclei is not known. The Egr2-Pet1 population of medullary raphe neurons appears to be responsible for the serotonergic contribution to the ventilatory response to CO_2_, and they have been reported to project to the medial PB^20^. Studies of serotonergic afferents to the lateral PB have found that they mainly originate in the DR^14–16^, but have not determined whether the terminal field involves the PBel. We therefore examined sections through the PBel in animals that received injections of AAV-Flex-ArchT-GFP into the DR of Sert-Cre mice (Fig. 6a–c). Anterogradely labeled axons from DR serotonergic neurons intensely innervated the PBel, with minimal innervation of the medial or dorsolateral PB (Fig. 6c). Similarly, after unilateral injection of cholera toxin subunit b (CTb) in the PB of the Sert-L10 mice, we observed retrogradely labeled serotonergic neurons predominantly in the DR with very few neurons seen in MR (*n* = 4; Supplementary Fig. 5a–c). Thus the area of the PBel that contains CGRP neurons appears to receive serotonergic input primarily from the DR.

### Inhibiting the terminal field of DR^Sert^ neurons in the PBel

Mice were injected in the DR (*n* = 18) with AAV-FLEX-ArchT-GFP and implanted with glass fibers targeting the PBel bilaterally (Fig. 6a–c). Eight of these mice were histologically verified to have expression of GFP in axons targeting the PBel and that the tips of the glass fibers were along the surface of the PBel. In these mice, during the Laser-ON condition the latency to arousal again was greatly increased to 34.9 ± 2.3 sec (F_1,14_ = 56.9; *P* < 0.001; Fig. 6d) and the animals failed to wake up to the CO_2_ within 30 sec in 43.8% of the trials (F_1,140_ = 16.63; *P* = 0.003; Fig. 6e and f) compared with the Laser-OFF condition, which had a latency of 16.6 ± 0.9 sec to arousal and showed no failures of arousal to CO_2_ within 30 sec. The effect of inhibition of the DR^Sert^ terminals in the PBel on the latency to arousal was comparable to, and was not significantly different from that after inhibition of the DR^Sert^ cell bodies (Fig. 6g–i). Hence, the main effect of the DR^Sert^ neurons on arousal to CO_2_ appears to be owing to their action on the PBel. In mice in which the tip of the glass fiber was 1–2 mm away from the PBel, the Laser-ON treatment had no effect on the arousal latency (medial PB, *n* = 4; arousal latency = 18.4 ± 1.1 sec; lateral PB *n* = 4; arousal latency=17.3 ± 1.6 sec).

### Is the effect of DR^Sert^ neurons on CO_2_ arousal mediated by 5HT_2a_ receptors in the PBel?

Given the restoration of CO_2_ arousal by a 5HT_2a_ receptor agonist in mice lacking serotonin neurons, we hypothesized that the DR^Sert^ effect on CO_2_ arousal was mediated by 5HT_2a_ receptors on the PBel^CGRP^ neurons.

**Fig. 7:**
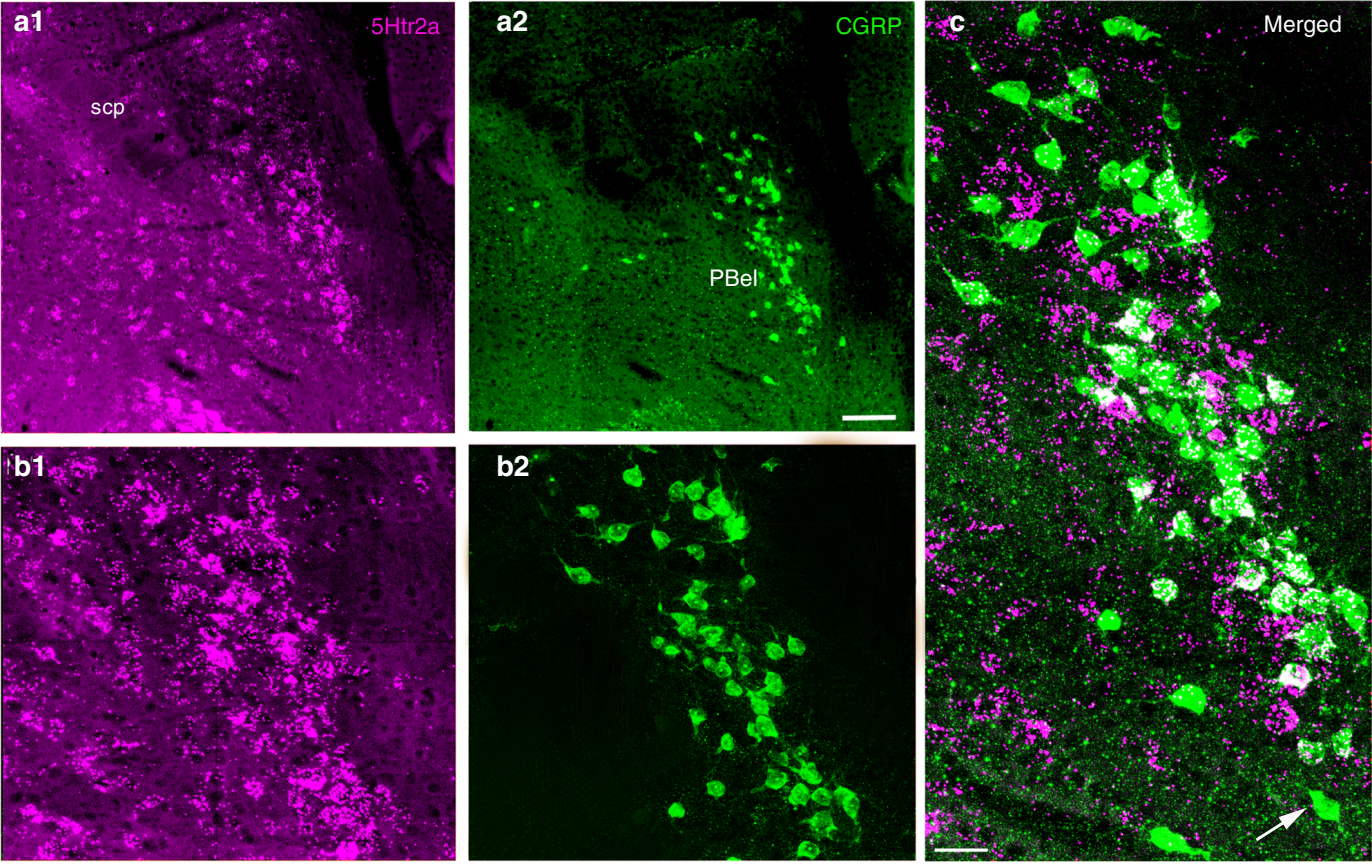
Expression of 5HT_2a_ receptors mRNA (5Htr2a) by PBel^CGRP^ neurons. Immunofluorescence photomicrographs of a brain section through the parabrachial area from a CGRP-L10-GFP mouse showing 5HT_2a_ receptor fluorescence in situ hybridization in magenta (**a1** and **b1** at low and higher mag) and CGRP neurons in green (**a2**, **b2**). The image is pseudo-colored in magenta-green to facilitate viewing by readers with color blindness. The merged image (**c**) shows that nearly all of the green CGRP neurons have some in situ hybridization grains (white) over their cell bodies (seen in *n* = 3). A lone exception is indicated by the white arrow. The scale bar represents 100 µm in **a1** and **a2**; 50 µm in **b1** and **b2**; and 20 µm in **c**. scp, superior cerebellar peduncle.

To investigate 5HT_2__a_ receptor expression in the PB, we used RNA scope in situ hybridization for the 5HT_2a_ receptor mRNA (5Htr2a) on sections through the PB in which the CGRP neurons express L10-GFP (Fig. 7). We found 5HT_2a_ receptor mRNA expression in many cell groups in the dorsolateral pons, including the central lateral, medial, and external lateral PB subnuclei. However, there was a dense accumulation of 5HT_2a_-expressing neurons in the PBel, which overlapped with the location of the PB^CGRP^ neurons. In doubly stained sections, we found that nearly all of the PB^CGRP^ neurons contained labeling for the 5HT_2a_ receptor. Notably, very few neurons lateral to the borders of the PB^CGRP^ group, in the lateral crescent region where the neurons are known to drive ventilation, expressed 5HT_2a_ mRNA.

**Fig. 8:**
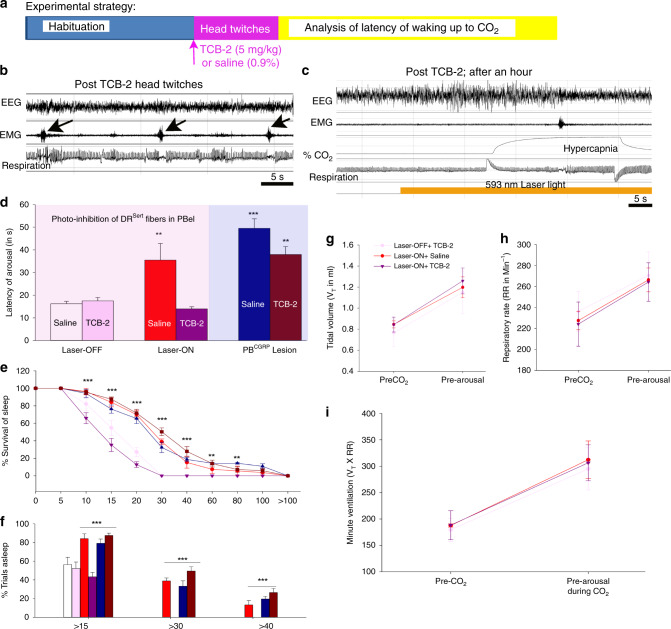
5HT_2a_ agonist reverses blockade of CO_2_ arousal caused by photoinhibition of DR^*Sert*^ terminals in the PBel, but not by deletion of PBel^*CGRP*^ neurons. Panel **a** shows the experimental strategy that involved 3 h habituation to the plethysmographic chamber followed by with intraperitoneal injections of either 0.9% saline or TCB-2 (5HT_2a_ agonist; 5 mg/Kg), and CO_2_ exposure either with photoinhibition of the DR^Sert^ terminals in the PBel or with deletions of the PBel^CGRP^ neurons. **b** is a representative polysomnographic record showing head twitches in the first hour after injection of TCB-2 (marked by arrows). To avoid confusion with arousal, this period was not used to analyze the latency of arousal to CO_2_. **c** shows a representative trial of CO_2_ exposure more than one hour after TCB-2 injection when animals attained stable sleep; data in this 3 h period were used for analyzing the latency to waking up to CO_2_. Graphs **d**–**f** show for each group the mean (± SEM) latency of arousal (**d**), survival curves (**e**), and percent of trials (mean ± SEM) in which the animal remained asleep at various time points after onset of CO_2_ exposure (**f**). **P < 0.01; ***P < 0.001; one-way (**d**) or two-way ANOVA (**e** and **f**). Silencing the DR^Sert^ terminals in PBel (Laser-ON) is compared here with the control (Laser-OFF), in both conditions (with injection of the 5HT_2a_ agonist- TCB-2 or saline; *n* = 3). Activation of 5HT_2a_ receptors completely reversed the blockade of hypercapnia-induced arousal caused by inhibiting DR^Sert^ terminals in the PBel (F_3,9_ = 8.05; *P* = 0.006 for Laser-ON-saline, *P* = 0.91 for Laser-ON TCB-2, one-way ANOVA followed by multiple comparisons of treatment groups). In the PBel^CGRP^ deletion mice (*n* = 5), the arousal to CO_2_ is compared between the saline and TCB-2 groups (F_5,17_ = 15.4; *P* < 0.001 for saline and *P* = 0.002 for TCB-2, one-way ANOVA followed by multiple comparisons), where TCB-2 is ineffective in restoring the hyperapnia-induced arousal in the absence of the PBel^CGRP^ neurons. Effect of TCB-2 (5HT_2a_ receptor agonist) on respiration: **g**–**i** The respiratory airflow signals prior to CO_2_ exposure and during CO_2_ but prior to EEG arousal were analyzed off-line for tidal volume (mean ± SEM) (**g**), respiratory rate (mean ± SEM) (**h**), and minute ventilation (**i**). Laser-ON with either TCB-2 or saline injection, or Laser-OFF with TCB-2, showed identical tidal volume, respiratory rate, and minute ventilation prior to and during CO_2_ exposure, indicating that neither inhibition of the DR^Sert^ terminals in the PBel nor restoring the CO_2_ arousal response with TCB-2 affects the ventilatory response to CO_2_. Data in **d**–**i** are presented as mean values ± SEM.

We therefore injected a group of mice (*n* = 3) with verified inhibition of CO_2_ arousal by photoinhibition of DR^Sert^ axons in the PBel, with TCB-2 (5 mg/kg), a selective 5HT_2a_ receptor agonist to determine whether it could restore CO_2_ responses. Because TCB-2 has previously been shown to cause head twitching for ~1 hr after a 5 mg/kg dose, we tested the mice over the period from 1–4 hours after drug injection (Fig. 8a–c). In this group of animals, treatment with TCB-2 reduced the latency to CO_2_ arousal in the Laser-ON condition from 35.48 ± 7.31 sec to 16.24 ± 1.06 sec (F_3,9_ = 8.05; *P* = 0.006; power of the test = 0.88) (Fig. 8d–f), and the number of trials with no arousal within 30 sec was reduced from 38.8% to 0% (F_1,4_ = 147; *P* < 0.001). In order to further confirm if TCB-2 is acting through the 5HT_2a_ receptors specifically via the PBel^CGRP^ neurons, we deleted PBel^CGRP^ neurons by injecting AAV-DTA bilaterally in the PB of the CGRPCreER (*n* = 5) mice, and then recorded them for arousal response to CO_2_ with injection of either saline or TCB-2 (5 mg/kg). We observed that, as expected, the mice with PBel^CGRP^ deletions had significantly higher latencies (49.5 ± 4.17 sec) compared with the control group with no lesions and Laser-OFF (F_5,17_ = 15.4; *P* < 0.001; Fig. 8d–f) and were similar to those with Laser-ON in the previous group (photoinhibition of DR^Sert^ in PBel). After TCB-2 injections the arousal latencies to CO_2_ in this group of animals remained high (38.0 ± 3.5 sec; Fig. 8d–f) with failures to wake up during CO_2_ in 26% of the trials. These values were not statistically different from the saline or Laser-ON groups, consistent with the TCB-2 predominantly acting on the PBel^CGRP^ neurons to facilitate the arousal response to CO_2_.

Finally, in previous studies, 5HT_2a_ receptor agonists had relatively little effect on ventilatory responses to CO_2_. We therefore compared the tidal volume, respiratory rate and minute ventilation in the groups injected with TCB-2 vs. vehicle in both the Laser-ON and Laser-OFF conditions, during our CO_2_ arousal paradigm. The ventilation was calculated during the last five breaths before onset of the CO_2_ stimulus, and for the last five breaths prior to arousal (in the Laser-OFF condition and the Laser-ON + TCB-2 condition). For trials in the Laser-ON + vehicle (Saline) condition in which the animals did not wake up during the CO_2_ stimulus, we calculated the ventilation during the last five breaths before the mean time to arousal in the Laser-OFF condition. We found no change in the respiratory rate, tidal volume, or minute ventilation in response to CO_2_ in the animals in the Laser-ON vs Laser-OFF condition or between the administration of TCB-2 or vehicle (Fig. 8g–i). Hence, although the DR^Sert^ input to the PBel^CGRP^ cell group appears to be necessary for EEG arousal to CO_2_, it does not appear to play a role in the respiratory response to CO_2_.

## Discussion

Our results show that the DR supplies serotonergic terminals to the PBel, and deletion of serotonergic neurons in the DR correlates significantly with the increases in the latency of arousal to CO_2_ (2.2-fold) with substantial numbers of animals failing to arouse during the 30 sec CO_2_ stimulation period (46%; with no control animals failing to arouse). Deletion of serotonergic neurons in the MR, on the other hand, does not show significant correlation to the latency to CO_2_ arousal, indicating that they play little if any role in the CO_2_ arousal response.

Optogenetic silencing of the DR^Sert^ neurons with ArchT resulted in a prolongation of the time to arousal by 2.96-fold and showed 40% failure to arouse, suggesting that acute inhibition of the DR^Sert^ neurons may have a more potent effect than chronic deletion (i.e., there may be some compensation in the chronic deletion animals), as both deletions and ArchT transfection affected similar proportions of the DR^Sert^ neurons.

Interestingly, neither the deletion of the DR^Sert^ neurons nor driving them with the hM3Dq receptor and CNO affected the baseline amount of wake–sleep. The AAV-DTA, however, only killed an average of ~80% of the DR^Sert^ neurons in each animal, and the AAV-hM3Dq transfected only about half of the neurons. Although it is possible that the remaining 20% of DR^Sert^ neurons could support wake–sleep states, it seems unlikely that activating half of them would not have any effect on these measurements. We think it is more likely that the DR^Sert^ neurons are not themselves arousing the forebrain, but that they have a modulatory effect on some other cell group(s) with potent arousal effects. In addition, the lack of effect of CNO on the latency to arousal suggests that serotonin is necessary for this downstream circuit to be effective, but that it has a ceiling effect as chemogenetic driving of the DR serotonin neurons does not further increase arousability beyond the normal range. (Alternatively, it is possible that the ceiling effect is owing to the lag in CO_2_ levels rising in the chamber during the trial, such that the CO_2_ sensory limb takes ~13–15 sec to sense the CO_2_, no matter how potent the downstream cortical arousal system is).

Next, we tested whether the effect of the DR neurons on CO_2_ arousal might be mediated by the selective DR serotonergic input to the PBel. We found that inhibiting the DR^Sert^ terminals in the PBel alone caused a 2.1-fold increase in the time to CO_2_ arousal, and a failure to arouse during the 30 sec of CO_2_ stimulation on almost 44% of trials. In other words, the bulk of the effect of the DR^Sert^ neurons on CO_2_ arousal appears to be mediated by their input to the PBel, whose CGRP neurons appear to provide the critical arousing influence to the forebrain.

Finally, we found that administration of a 5HT_2a_ receptor agonist restored CO_2_ arousal even while the DR^Sert^ terminals in the PBel were photoinhibited. Although it is possible that the 5HT_2a_ agonist may have been acting at other sites as well, it is unable to restore CO_2_ arousal when the PBel^CGRP^ neurons are deleted. Hence, the release of serotonin by DR terminals in the PBel is critical for CO_2_ arousal, although we cannot determine whether the response is owing to 5HT_2a_ receptors expressed by PBel neurons or perhaps presynaptically on a critical afferent to them. In addition, neither the photoinhibition of the DR serotonergic terminals in the PBel nor the 5HT_2a_ agonist had any effect on CO_2_ ventilatory response, indicating that the effects of serotonin on CO_2_-induced increases in ventilation and cortical arousal are carried by different circuits.

Although several studies have reported that serotonergic neurons in the DR are most active during waking and least active during REM sleep^21–23^, these recordings were extracellular and depended upon the distinctive action potential morphology of monoamine neurons to determine which were serotonergic. More recent studies, however, have emphasized that the DR contains a mixed population of dopaminergic and serotonergic neurons, as well as GABAergic and glutamatergic neurons, with distinct electrophysiological characteristics and functional relevance^22,24–28^. In particular, it would be difficult to distinguish DR^Sert^ neurons from DR dopamine neurons, which are known to have a wake-active profile,^24,25,29^ using extracellular recordings. Studies examining the effects on sleep of manipulation of the serotonin system have in the past been confounded by the role played by the serotonin system in thermoregulation^10,30–32^ and by the difficulty in manipulating just the DR serotonin neurons. Recently, Ito and colleagues used optogenetic stimulation of DR neurons in tryptophan-hydroxylase-ChR2 mice, finding that this increased wake and decreased NREM sleep during 1 hr of stimulation at 20 Hz^33^. However, our experiments employing selective AAV-DTA deletions or hM3Dq activation of only the DR^Sert^ neurons found no effect on baseline wake–sleep. There are several possibilities for this discrepancy. One is that the stimulation rate used by Ito and colleagues may be outside the physiological firing range for DR neurons, which typically fire at ~3 Hz during quiet wake, but were stimulated at 20 Hz optogenetically^34^. Another possibility is that Ito et al. used Tph-Chr2 mice that express channelrhodopsin in all serotonergic neurons. Hence, depending upon their optical fiber placement, they may have also been stimulating median raphe neurons which are just ventral to the DR (and which did not show hM3Dq expression in our experiments). Alternatively, because we were able to transduce fewer than 50% of DR^Sert^ neurons with the hM3Dq vector, it is possible that the effect of stimulating DR serotonin neurons may be subtle unless a large proportion of the population is activated at the same time.

Regardless of their role in baseline wake–sleep, serotonin neurons clearly have a very important role in arousal during hypercapnia. Lmx1b^f/f/p^::EPet1-Cre mice with deletion of nearly all the serotonergic neurons have impaired arousal to CO_2_^10^. However, deletion of medullary serotonin neurons in Egr2-Pet1 mice reduces the ventilatory response to CO_2_, suggesting that the medullary serotonin neurons innervate and facilitate the medullary cell groups that promote ventilation, but not the ascending arousal system. Because both medullary and DR serotonin neurons are known to be CO_2_ responsive^8,9^, and DR neurons were previously found to target lateral PB^14–16^, it therefore seemed likely that the DR neurons may contribute to CO_2_ arousal by means of their projections to the PB. A recent study supported this idea by showing that stimulation of the DR by microinjection of acidic cerebrospinal fluid woke up wild-type mice, but not Lmx1b^l/fl/p^::ePet1-Cre mice that lack serotonin neurons^17^. Our results are consistent with these earlier findings, by showing that either selective deletion or acute photoinhibition of the DR^Sert^ neurons increases the latency and reduces the frequency of CO_2_ arousals. We observed a smaller effect on the arousal latency after attempts to delete MR^Sert^ neurons, which are also chemosensory^35^. However, while there was also loss of ~20% of DR^Sert^ neurons in these experiments (owing to leakage of the viral vector back along the needle track), the effect on latency to CO_2_ arousal correlated with the loss of DR^Sert^ but not MR^Sert^ neurons in these experiments.

DR^Sert^ neurons regulate CO_2_-induced arousals by acting on 5HT_2a_ receptors^13^, and activation of 5HT_2a_ receptors restores CO_2_ arousal in Lmx1b^f/f/p^::ePet1-Cre mice^10^. Our anatomical data show that the DR^Sert^ neurons provide an intense projection to the PBel, the site that contains the CGRP neurons which we previously showed to be necessary for CO_2_ arousal. We therefore tested the role of the projection from DR^Sert^ neurons to PBel in causing CO_2_ arousal. Inhibition of the DR^Sert^ neuronal terminals in the PBel produced blockade of CO_2_ arousal responses similar in proportion to that seen with inhibition or deletion of the DR^Sert^ cell bodies. These findings suggest that the effects of the DR^Sert^ neurons on CO_2_ arousal are mediated almost entirely by their projections to PBel^CGRP^ neurons. Because directly deleting the DR^Sert^ neurons had no effect on baseline wake–sleep, and driving them with the hM3Dq receptor did not cause wakefulness or affect CO_2_ arousal, it is likely that the DR^Sert^ neurons under normal physiological conditions play a mainly modulatory role, by sensitizing the PBel^CGRP^ neurons to other CO_2_-responsive inputs (e.g., from the retrotrapezoid nucleus or nucleus of the solitary tract). Thus while stimulation of the DR by infusion of acidic cerebrospinal fluid using an in vivo dialysis cannula with a tip length of 1 mm woke up wild-type mice in a little over 30 sec^17^, it is difficult to assess the extent to which the acid stimulus may diffuse through the brain and the CSF. Exposure to increased inspired CO_2_ typically awakens mice in <15 sec in our experiments, so the diffusion of the acid from the dialysis cannula in 30 sec may have activated both the DR neurons and the RTN neurons, which are ~2.5 mm away. We further observed that the effect of inhibition of DR^Sert^ terminals in the PBel was reversed by injection of the 5HT_2a_ receptor agonist TCB-2, suggesting that DR neurons activate PBel^CGRP^ neurons by acting on 5HT_2a_ receptors^36^.

To determine whether the PB^CGRP^ neurons themselves express the 5HT_2a_ receptor, we used in situ hybridization to demonstrate that they express 5HT_2a_ receptor mRNA. Interestingly, 5HT_2a_ receptor mRNA was not expressed by neurons lateral to the PB^CGRP^ group, in the lateral crescent area, where the neurons project to the ventrolateral medullary respiratory cell groups and stimulation is known to cause increased ventilation^37^. These results were consistent with TCB-2, a 5HT_2a_ receptor agonist, restoring arousal responses to CO_2_, but having little if any effect on ventilatory responses. Although it is possible that TCB-2 may act presynaptically, e.g., on terminals of afferents to the PB^CGRP^ neurons to sensitize them to CO_2_ sensory inputs, it appears that the direct effect of serotonin on the PB^CGRP^ neurons is mediated at least in part by 5HT_2a_ receptors directly on the target neurons themselves. The degree to which PB^CGRP^ neurons may express other 5HT receptors, or to which the 5HT_2a_ effect on them may be presynaptic, will require further study.

In summary, our findings reconcile two lines of work on the role of the PBel^CGRP^ neurons and the serotonin system in CO_2_ arousal. The EEG arousal is apparently caused by inputs from CO_2_-responsive neurons, in the retrotrapezoid nucleus (central CO_2_ sensors) and nucleus of the solitary tract (from peripheral CO_2_ sensors), to the PBel^CGRP^ neurons, which are required for the awakening response. Our previous work shows that the forebrain arousal depends upon projections to the basal forebrain^7^, where new work finds that activation of parvalbumin-expressing GABA neurons causes EEG desynchronization to hypercapnia^38^. This response requires DR^Sert^ neurons to provide a level of serotonergic input to the PBel acting on 5HT_2a_ receptors that sensitizes them or modulates their response. Loss of either the PBel^CGRP^ neurons or their serotonergic input prevents CO_2_ awakening. However, the ventilatory response to elevated CO_2_ appears to rely upon other circuits, and while it also requires serotonin, this is not from the DR and it is not mediated by 5HT_2a_ receptors. These observations suggest that it may be possible to modulate the EEG arousal separately from the ventilatory response to elevated CO_2_, which could potentially allow a patient with sleep apnea to keep the airways open, whereas avoiding the EEG arousals that result in sleep fragmentation, cognitive impairment, and metabolic and cardiovascular diseases.

Finally, recent studies suggest that the PBel^CGRP^ neurons may respond to a wide array of visceral stimuli, associated with gastrointestinal disturbance, pain, conditioned taste avoidance, and conditioned fear responses^39,40^. It is currently not known whether the PBel^CGRP^ neurons that respond to these other inputs also require 5HT_2a_ receptor input from the DR. If so, 5HT_2a_ inhibition may be a way to suppress sleep fragmentation caused by pain, gastrointestinal disturbances, and even post-traumatic stress disorder.

## Methods

### Animals

Our studies employed the *Slc6a4* transgenic BAC-Cre-recombinase driver line, which selectively expresses Cre-recombinase in^41^ serotonin neurons (Sert-Cre). All transgenic mice used here were heterozygous for the transgene and backcrossed to the C57BL6 strain and wild-type littermates were used as controls. All mice used in each experiment are derived from at least three different litters. We validated the presence of the Cre-recombinase enzyme in these mice, by immunostaining the brain sections from the F1 progeny of adult male Sert-Cre (*n* = 4) mice crossed with a R26-lox-STOPlox-L10-GFP “Cre-reporter” also referred to as Sert-Cre::L10-GFP or SERT-L10^42^ (Fig. S1), for GFP and serotonin, and found 97.8 ± 0.5% of the green cells (Cre containing) were colocalized with serotonin and 96.8 ± 0.5% of the serotonin cells were colocalized with Cre-recombinase/GFP (also shown by Gong et al.^41^), indicating that these mice can be reliably used for selective manipulation of DR^Sert^ neurons. We bred these mice in our animal facility and confirmed their genotype by using a Red Extract N-amp Tissue PCR kit (Sigma-Aldrich; Catalog # XNAT-1000RXN) and Cre forward and reverse primers to detect the Cre-recombinase gene. Their wild-type litter mates were used as controls, in each experiment.

We also used CGRPCreER mice (*n* = 8) for two studies mentioned in Experiment 5. In this experiment, CGRPCreER (*n* = 5) were injected bilaterally in the PB by AAV-Flex-DTA to cause cre-dependent killing of the CGRP neurons as done previously by us^7^.

All mice used in these experiments were male. Animals were maintained on a 12 h light/dark cycle with ad libitum access to water and food and were singly housed after surgery, with ambient temperature of 21–23 ^o^C and humidity levels between 40 and 60%. Male littermates were randomly assigned to the experimental groups. All animal procedures met National Institutes of Health standards, as described in the Guide for the Care and Use of Laboratory Animals, and all protocols were approved by the Beth Israel Deaconess Medical Center Institutional Animal Care and Use Committee.

### Vectors

An AAV conditionally expressing subunit-A of diphtheria toxin in a Cre-dependent manner (AAV-Flex-DTA) was prepared by packaging the DTA gene into a FLEX cassette. Within the FLEX cassette the DTA sequence is inverted, and as such it cannot be transcribed except in the presence of cre-recombinase, conferring absolute expression selectivity. This construct also contained mCherry, with mCherry located external to the FLEX switch. Hence mCherry was expressed in all non-Cre-recombinase cells within the injection field (Fig. 1c), thereby allowing us to ascertain both the anatomic extent of the injection, and demonstrate, quantitatively, “survival” of the non-cre-recombinase cells intermingled with the Cre-recombinase cells targeted by DTA. This vector was designed, produced, and validated by Drs. Patrick M. Fuller and Michael Lazarus and has been used by us previously to selectively kill the CGRP neurons in the CGRPCreER mice^7^. The AAV containing the gene construct FLEX-hM3Dq-mCherry-wpre was also acquired from Dr. Patrick M. Fuller, and had been previously used to selectively activate cell populations in the basal forebrain and in the PB^7,43,44^.

For optogenetic experiments, we used the optogenetic neural silencer AAV-CAG-FLEX-ArchT-GFP (AAV-serotype-8) that co-expresses ArchT and GFP in a Cre-dependent manner. This viral vector was procured from the University of North Carolina (UNC) vector core and has been previously used for silencing neurons and their terminals by us^7^ and also by many other groups^45–47^. In order to test the Cre-dependent expression of the silencer AAV-CAG-FLEX-ArchT-GFP, we injected this into the DR of mice and found expression of ArchT (as shown by GFP) only in serotonin-expressing neurons (Fig. 4c1 and c2).

### Generation of AAV

The detailed process for the generation of the AAV-FLEX-DTA, AAV (serotype-10) has been described previously^7^. The viral vector expressing inhibitory opsin, ArchT, AAV-CAG-FLEX-ArchT-GFP was packaged at the UNC vector core^7^.

### Surgery

*Experiment 1: Selective deletion of DR*^*Sert*^ *neurons*: For deletion of the DR^Sert^ neurons, we injected a Cre-dependent viral vector expressing diphtheria toxin subunit-A (AAV-FLEX-DTA) in the DR (AP: −4.9 mm; DV: −2.5 mm; ML: 0.0 mm) of wild-type (WT; *n* = 6) and Sert-Cre-L10 mice (*n* = 13). Sert-Cre-L10 mice express GFP in all the serotonin and Cre expressing cells (Supplementary Fig. 1). The lesions in these mice were seen by loss of serotonin or GFP expressing cells. We counted the surviving GFP cells in these mice, by immunostaining the brain sections for the GFP at the end of the experiment and counting all immunostained neurons in three sections through the DR/MR. Cell counts were corrected for cell size using the Abercrombie correction factor. These mice were instrumented for sleep with implantation of EEG and EMG electrodes. Five weeks post injection of the viral vector (to ensure optimal expression of the gene cell deletion^7^, we recorded 24 h sleep–wake in the mice after acclimatizing them to the recording apparatus for a week. After sleep recordings, we recorded the arousal responses to CO_2_ by placing them in the plethysmograph and recording them for both sleep and breathing by the procedure described previously^7,18^.

*Experiment 2: Chemogenetic activation of DR*^*Sert*^ *neurons:* For activation of the DR^Sert^ neurons, we injected a Cre-dependent viral vector expressing hM3Dq in the DR (AP: −4.5 to −4.9 mm; DV: −2.5 mm; ML: 0.0 mm) of the Sert-Cre mice (*n* = 6). These mice were instrumented for sleep and recorded for the arousal responses to CO_2_ after intraperitoneal injections of either saline or CNO (0.3 mg/Kg), by the procedure described previously^7,18^.

*Experiment 3: Optogenetic inhibition of the DR*^*Sert*^ *neurons:* For selective inhibition of DR^Sert^ neurons, we injected an adeno-associated viral vector expressing the inhibitory opsin AAV-FLEX-ArchT-GFP in the DR (AP: −4.5 to −4.9 mm; DV: −2.5 mm; ML: 0.0 mm) and implanted these Sert-Cre mice (*n* = 10) with an optical fiber targeting the DR. We also injected some wild-type mice (*n* = 3) with AAV-FLEX-ArchT-GFP as well, which served as control. We recorded these mice for recording the arousal responses to CO_2_ by procedures mentioned earlier^7,18^.

Upon examining the sections through the PBel in animals with injections of AAV-FLEX-ArchT-GFP into the DR of Sert-Cre mice, we found intense innervation of the external lateral part of the parabrachial (PBel) area. To further confirm that PBel receives innervation from serotonergic neurons in the DR, we also injected Sert-L10 mice (*n* = 4) with retrograde tracer, cholera toxin subunit b (24 nl, 0.2% - CTb, List Biological Laboratories Inc.) unilaterally in the lateral PB. One week later these mice were sacrificed, brains perfused, and tissue sections processed for immunohistochemistry for CTb using the procedure described below.

*Experiment 4: Inhibiting the terminal field of DR*^*Sert*^ *neurons in the PBel:* A separate set of mice were injected in the DR with AAV-FLEX-ArchT-GFP (*n* = 8), and to test whether DR^Sert^ neurons mediate CO_2_ arousals by their input to the PBel, these mice were bilaterally implanted with optical fibers targeting the PBel (AP: −5.1 to −5.3 mm; DV −2.6 mm; ML: ±1.3 mm) for the inhibition of the DR^sert^ terminal fields. These mice were recorded for sleep and breathing at 5 weeks post injection in plethysmographs where the arousal responses to CO_2_ were assessed.

*Experiment 5: Inhibiting the terminals fields of DR*^*Sert*^ *neurons in the PBel in the presence of a 5HT*_*2a*_ *receptor agonist:* In six mice, we injected AAV-FLEX-ArchT-GFP in DR and implanted bilateral optical fibers in the same way as experiment 4, and recorded them for sleep and breathing in the Laser-ON condition. These experiments were conducted after intraperitoneal injection of either saline or 5HT_2a_ receptor agonist (TCB-2; at 5 mg/kg). TCB-2 (Tocris Bioscience, Ellisville, MO) was dissolved in sterile double-distilled water, aliquoted and stored at 4 ^o^C, until the time of systemic injection in mice. TCB-2 has been previously shown to be a selective serotonergic agonist at central 5HT_2a_ receptors when injected intraperitoneally^13,48^. To confirm whether 5HT_2a_ receptors are expressed on the CGRP neurons in the PBel, we crossed CGRPCreER mice with tD tomato reporter mice (*n* = 3), treated the progeny with tamoxifen (75 mg/kg) to activate the tD tomato reporter, and then 1 week later we immunostained brain sections with antibody against 5HT_2a_ receptors. To further assess, if TCB-2 is acting through the PBel^CGRP^ neurons, we deleted PBel^CGRP^ neurons in CGRPCreER (*n* = 5) mice, and recorded them for arousal response to CO_2_ either with saline or TCB-2 injection.

### Data acquisition

Mice were attached to the recording cables and acclimatized to the recording chamber for a week, before recording sleep in them for 24 h. All recordings were done at 5 weeks after injection of the viral vectors. All recordings during CO_2_ stimulation were done in a plethysmographic chamber (unrestrained whole-body plethysmograph, Buxco Research Systems) allowing us to record the breathing of the mouse while in the chamber and to document the gas mixture. Electroencephalogram (EEG) and electromyogram (EMG) were recorded using Pinnacle preamp cables connected to the analog adaptor (8242, Pinnacle Technology). Gas levels in the chamber were continuously monitored using CO_2_ and O^2^ monitors from CWE, Inc (Ardmore, PA, USA). EEG, EMG, respiration, and CO_2_ and O2 levels were fed into an Axon Digidata 1322 A analog-to-digital converter and the signals were acquired using Axoscope software- v10 (Molecular Devices, Foster City, CA, USA).

*Experiment 1:* EEG/EMG were recorded for 24 h after a week of acclimatization to the recording apparatus. Sleep recordings were done using a preamplifier connected to a data acquisition system (8200-K1-SE) and Sirenia Software v2.1 (from Pinnacle Technology). Twenty-four hours sleep–wake was compared between the WT and Sert-Cre mice with DR^sert^ deletions.

*Experiments 2–5:* Mice were connected to cables both for sleep recording as well as for transmitting laser light through the pre-implanted glass fiber (except for in Experiment 2 where stimulation was done using intraperitoneal injection of ligand CNO and compared with saline), and were placed in the plethysmographic chamber beginning at 9:00 A.M. for 4 h on each test day. They also then received one of the following protocols in a random order. Each of these protocols were repeated for each mouse for 2 days; on one of the days the laser was switched on (Laser-ON) and on the other day the laser was off (Laser-OFF), again in random order. On the Laser-ON protocol, a 593 nm laser was ON for 60 s followed by 5 mins off, and this was repeated 20 times per session. In the Laser-OFF condition, everything was the same, except that the laser light was not turned on. Twenty seconds after the scheduled onset of the laser, the gas intake for the plethysmograph was switched either to normocapnic air (21% O_2_, 79% N_2_) or hypercapnic air (10% CO_2_, 21% O_2_, and 69% N_2_) for 30 sec. Trials were analyzed for latency to arousal only for those epochs where the mouse was in NREM sleep for at least 30 s before the stimulus onset.

*Laser light:* Mice were allowed at least 2 d to acclimate to fiber optic cables (1.5 m long, 200 μm diameter; Doric Lenses, Quebec, QC, Canada) and connecting interfaces coated with opaque heat-shrink tubing before the experimental sessions. During Laser-ON experiments, light pulses were programmed using a waveform generator (Agilent Technologies, catalog #33220 A, CA, USA) to drive an orange–yellow light laser (593 nm; Laser Glow, Toronto, ON, Canada) to be on for 60 s beginning 20 s before the onset of the CO_2_ (or air or acoustic) stimulus. We adjusted the laser such that the light power exiting the fiber optic cable was 8–10 mW, and this was checked before and after the experiment. Using an online light transmission calculator for brain tissue (www.stanford.edu/group/dlab/cgi-bin/graph/chart.php), we estimated the light power at the PBel to be <10 mW/mm^2^ and a similar range has been used by most researchers for neuronal silencing at the terminal fields^45–47^. Note that this is probably a high estimate because some light is probably lost at the interface between the fiber optic cable and the implanted optic-fiber.

### Data analysis

*Sleep analysis*: Digitized polygraphic data were analyzed off-line in 10 s epochs using Sleep Sign software R. 3.3 (Kissei Comtec Co. Ltd., Matsumoto, Nagano, Japan). The software autoscored each epoch using an algorithm that identified three behavioral states based on EEG and EMG. The autoscored data were then checked at least twice visually for movement and any other artifact and to correct automatic state classification by an unbiased scorer blind to the treatment groups (MAK and RCT). Over-reading of the sleep recordings were done according to previously published criteria (Neckelmann and Ursin, 1993; Kaur et al., 2008). The changes in amount of time spent in different sleep–wake states and the latency of waking up to CO_2_ in different treatment groups were compared statistically using either one-way or two-way ANOVA (when compared over a period of time over light and dark phases) followed by a Holm–Sidak post hoc test for multiple comparisons.

*Statistical analysis*: All statistical analyses were performed using SigmaPlot 12.3 (Systat Software, Inc.). For statistical comparisons, we first confirmed if the data meet with the assumptions of the ANOVA, then either one-way or two-way ANOVA was performed to compare the effects between various treatment groups during the light and dark phase. If differences in the mean values among the treatment groups were greater than would be expected by chance; then all pairwise multiple comparisons were performed using the Holm–Sidak method. The *F* and *P* values are described in the results section with details of the statistical tests also given in the respective figure legends and represented in figures. The ‘*n*’ is reported in the figures and results and represents the number of animals, and the error bars represent mean ± SEM. Using SigmaPlot 12.3, we also tested the sample size and power of the tests post hoc and found that the power of each statistical test was at least 80% at alpha =  0.05, suggesting adequate sample sizes for all the experiments. A probability of error of <0.05 was considered significant.

*Analysis of arousal to hypercapnia*: EEG arousals in response to CO_2_ were identified by EEG transition from NREM to a waking state, which was usually accompanied by EMG activation, as described previously^7,18^. The duration and latency of all the EEG arousals after onset of stimulation were scored. The trials in which animals did not awaken during the 30 s of the CO_2_ or air stimulus were marked as failure to arouse to stimulus. These arousals were compared across the Laser-ON and Laser-OFF days.

*Histology*: At the conclusion of the experiment, the animals were perfused with 0.9% saline followed by 10% buffered formalin while under deep anesthesia. Brains were harvested for analysis of the effective location of the injection site. Brains were kept in 30% sucrose for 2 d and sections were cut at 30 μm using a freezing microtome in four 1:4 series.

*Immunohistochemistry*: In mice injected with ArchT, one series of sections was immunostained for GFP (Rabbit anti-GFP, 1:10 K, ThermoFisher Scientific, Cat- A11122) using standard immunohistochemistry protocols described previously^18^. Another series was double-stained for immunofluorescence with antibodies using GFP using mouse anti-GFP (Rabbit anti-GFP, 1:10 K, ThermoFisher Scientific, Cat- A11122; RRID:AB_10073917) and also stained with serotonin antibody (Rabbit anti-Serotonin, ImmunoStar, 1:5 K, Cat- 20080, RRID: AB_572263). Neither of these antibodies showed immunostaining when the primary antibodies were omitted, and with GFP when the tissue from control mice was used that were not injected with viral vector. Some of the brains (*n* = 4) from Sert-L10 mice injected with CTb were immunostained using Goat anti CTb (1:30 K, Cat# 703, RRID:AB_10013220, List Biological Laboratories Inc., CA).

In brief, all the sections for immunostaining were first incubated in 0.1 m phosphate buffer and 1% H_2_O_2_ for 5–10 min followed by three washings in 0.1 m phosphate buffer. For all the immunohistochemical staining that involved visualization using a diaminobenzidine (DAB) reaction, the sections after the overnight incubation with primary antiserum were incubated in the respective secondary antibodies for 2 h, followed by incubation in ABC reagents (1:1000; Vector Laboratories) for 90 min, then washed again and incubated in a 0.06% solution of 3,3-DAB tetrahydrochloride (Sigma-Aldrich) in phosphate-buffered saline (PBS) plus 0.02% H_2_O_2_ for 5 min. Finally, the sections were mounted on slides, dehydrated, cleared, and cover-slipped. Sections for double staining for GFP, Serotonin, CTb or 5HT_2a_ were incubated in fluorescent-labeled secondary antibodies (Alexa- 488 at 1:200 or Alexa- Cy3 at 1:200; Catalog #- A32790 and A10521, RRID- AB_2762833 and RRID- AB_2534030, Molecular probes, Thermo-Fischer Scientific) for 2 h and cover-slipped with fluorescence mounting medium (Dako, North America).

*Histological analysis*: To analyze the deletion of the DR^sert^ and MR^Sert^ neurons (Fig. 2d and e), we counted the serotonin cells in three sections at three levels of DR separated by 300 µm, in both the Sert-Cre-L10 mice (*n* = 13) with AAV-DTA injections and in *n* = 3, we used the Sert-Cre-L10, with no injections, where the GFP fluorescence shows up in all the serotonin-Cre positive cells (Supplementary Fig. 1). The counts for DR were conducted using a boundary box (2 mm × 1 mm), centered on the midline above the decussation of the superior cerebellar peduncle and included the lateral parts of the DR^Sert^ population as well. For the median raphe the serotonergic cells below the decussation were counted in boxed area of 0.3 mm × 0.6 mm also centered on the midline. Only profiles with a clear nucleus were counted by an unbiased scorer (MAK and RCT), and the diameters of 20 nuclei were measured from each group, and the Abercrombie correction factor applied^18,49,50^.

*In situ hybridization (RNA Scope)*: We used CGRPCreER mice crossed with R26-lox-STOPlox-L10-GFP reporter mice (CGRP-L10, *n* = 3), for labeling 5Htr2a mRNA in the brain sections from PB area using RNA scope in situ hybridization. The brain was sectioned at 30 µm and mounted on glass slides in RNAase-free conditions, and RNA scope was performed using the multiplex fluorescent reagent Kit V2 (Cat# 323100, Advanced Cell Diagnostics, Hayward, CA). Brain sections on the slides were pretreated with hydrogen peroxide for 20 min at room temperature and then with target retrieval reagent for 5 minutes (at temperature above 99°C), followed by dehydration in 90% alcohol and then air-dried for 5 minutes. This is followed by a treatment with protease reagent (Protease III) for 30 minutes at 40°C. After rinsing in sterile water, sections were hybridized in 5Htr2a-C1, RNA scope probe (Mus musculus 5-hydroxytryptamine (serotonin) receptor 2a (Htr2a); Cat# 401291, Advanced Cell Diagnostics) for 2 hours at 40°C, and this probe has been used previously to selectively label 5HT_2a_ receptors on the brain tissue^51^. Sections were then incubated in three amplification reagents (AMP) at 40°C (AMP1 for 30 minutes, AMP2 for 30 minutes and AMP3 for 15 minutes) followed by Horse radish peroxidase—C1amplification at 40°C for 15 minutes. Sections were then incubated in tyramide signal amplification (TSA) reagents with Cy3 fluorophore (Cat# NEL744001KT, Perkin Elmer, 1:800) for 30 min to amplify and visualize 5Htr2a mRNA. In the final step, sections were subjected to HRP blocking for 15 min at 40°C. After each step, sections were washed with 1× wash buffer provided in the kit. Following the 5Htr2a RNA scope in situ hybridization, immuno-labeling of GFP was performed on the same sections, as the in situ procedure quenches the GFP fluorescence. For this, the brain sections were incubated in Rabbit anti-GFP (1:7500), (Cat#A6455;Lot#1220284; Molecular probes;) for overnight at 4 °C, washed in PBS (3×2 minutes) and then incubated in secondary antibody (Alexa Fluor- 488 Donkey anti Rabbit, Life Technologies, Cat# A-21206) for 2 h at room temperature. Finally, the slides were dried and cover-slipped with Dako fluorescence mounting medium (Cat# S302380-2, Agilent, CA).

*In vitro electrophysiological recordings*: For in vitro electrophysiological recordings we used *Sert-Cre* mice (*n* = 2). We injected 200–250 nl of AAV-FLEX-ArchT-GFP into the dorsal raphe nucleus (DRN; mid-caudal) using the coordinates described above. Four to five weeks after AAV injections, we prepared coronal slices for electrophysiological recordings using the same methods previously reported^52^. We recorded ArchT-GFP expressing neurons in the DRN using a combination of fluorescence and infrared differential interference contrast microscopy. We recorded in whole-cell or cell-attached configurations using a Multiclamp 700B amplifier, a Digidata 1322 A interface, and Clampex 9.0 software (Molecular Devices, Foster City, CA, USA). Neurons showing over time changes in input resistance >10%, were excluded from the analysis. We photoinhibited the ArchT-GFP expressing neurons in DRN using full-field light openings (~3 mW/mm^2^, 1 mm beam width) from a 880 mW LUXEON light-emitting diode (565 nm wavelength; #M565L3; Thorlabs, Newton, NJ, USA) coupled to the epifluorescence pathway of microscope. We recorded using a K-gluconate-based pipette solution, in whole-cell current clamp mode or in cell-attached configuration (*V*_h_ = 0–5 mV). The ACSF solution contained (in mM): 120 NaCl, 2.5 KCl, 1.3 MgCl_2_, 10 glucose, 26 NaHCO_3_, 1.24 NaH_2_PO_4_, 4 CaCl_2_, 2 thiourea, 1 Na-L-ascorbate, 3 Na-pyruvate (pH 7.3–7.4 when carbogenated with 95% O_2_ and 5% CO_2_; 310–320 mOsm). The K-gluconate-based pipette solution contained (in mM): 120 K-Gluconate, 10 KCl, 3 MgCl_2_, 10 HEPES, 2.5 K-ATP, 0.5 Na-GTP (pH 7.2 adjusted with KOH; 280 mOsm). In all the recordings we added 0.5% biocytin in the pipette solution to mark the recorded neurons. After in vitro recordings, we fixed the recorded slices in 10% buffered formalin (overnight) and then we placed them for 12–24 hours in streptavidin-conjugated Alexa Fluor AF-555 (orange–red; 1:500; Invitrogen) to fluorescently label the recorded neurons filled with biocytin. We imaged the biocytin labeled neurons using a Zeiss LSM 5 Pascal confocal microscope using Zen 2009 software (Zeiss). We analyzed the in vitro recording data using Clampfit 10 (Molecular Devices), MiniAnalysis 6 software (Synaptosoft, Leonia, NJ, USA) and MathLab (MathWorks; Natick, MA, USA) software. Figures were generated using Igor Pro 6 (WaveMetrics), Prism 7 (GraphPad, La Jolla, CA, USA), and Photoshop (Adobe) software. We calculated firing frequency and membrane potential changes by comparing values before, during and after light stimulation. We represented data as mean ± SEM and *n* refers to the number of cells. We compared group means using one-way ANOVA repeated measures followed with Holm–Sidak’s multiple comparisons post hoc test. Values showing *p* < 0.05 were considered significant.

### Reporting summary

Further information on research design is available in the Nature Research Reporting Summary linked to this article.

## Supplementary information

Supplementary Information

Reporting Summary

## Data Availability

All data generated to support the findings of this study are available from the corresponding author upon reasonable request.
