# Supplementary material for: Role of serotonergic dorsal raphe neurons in hypercapnia-induced arousals
Source: Nat Commun. 2020 Jun 2;11:2769. doi: 10.1038/s41467-020-16518-9 (PMC7265411; doi:10.1038/s41467-020-16518-9)
Supplement: Supplementary file 1 — Supplementary Information [file 41467_2020_16518_MOESM1_ESM.pdf]

# Role of Serotonergic Dorsal Raphe Neurons in Hypercapnia-Induced Arousals

Kaur et al.,

**Supplementary Figures:**

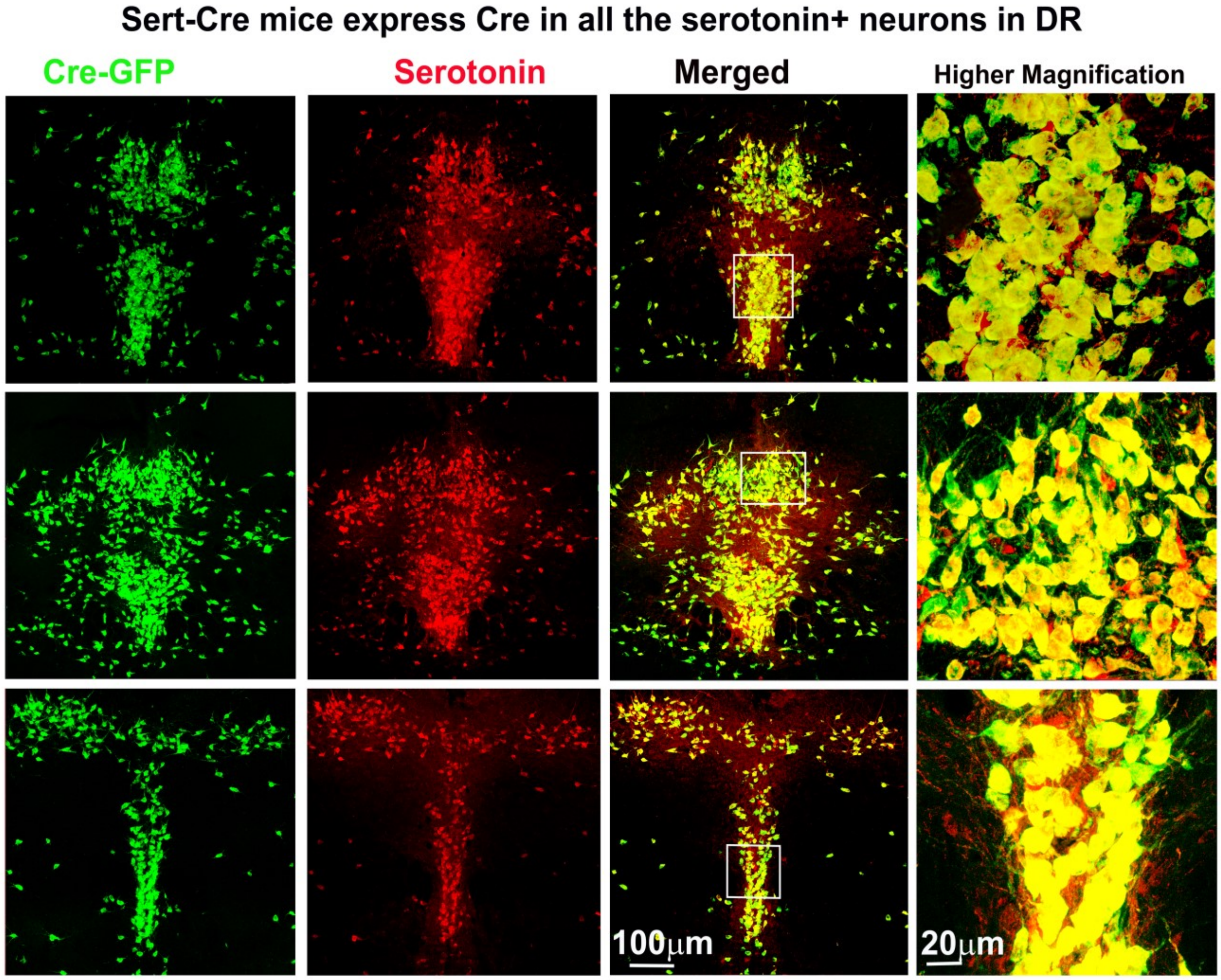


**Supplementary Figure 1** Validation of Cre expression in all the serotonin neurons in DR: We validated the presence of the Cre-recombinase enzyme in adult male Sert-Cre mice by crossing them with R26-lox-STOPlox-L10-GFP mice which express GFP in the ribosomes of serotonergic neurons. Brain sections were immunostained for GFP (first column) and serotonin (second column), with the third and fourth column showing the merged photomicrographs at low and high magnification. A near complete co-localization of both was seen in most sections confirming the presence of Cre-recombinase only in serotonin neurons (seen in n=4 mice).

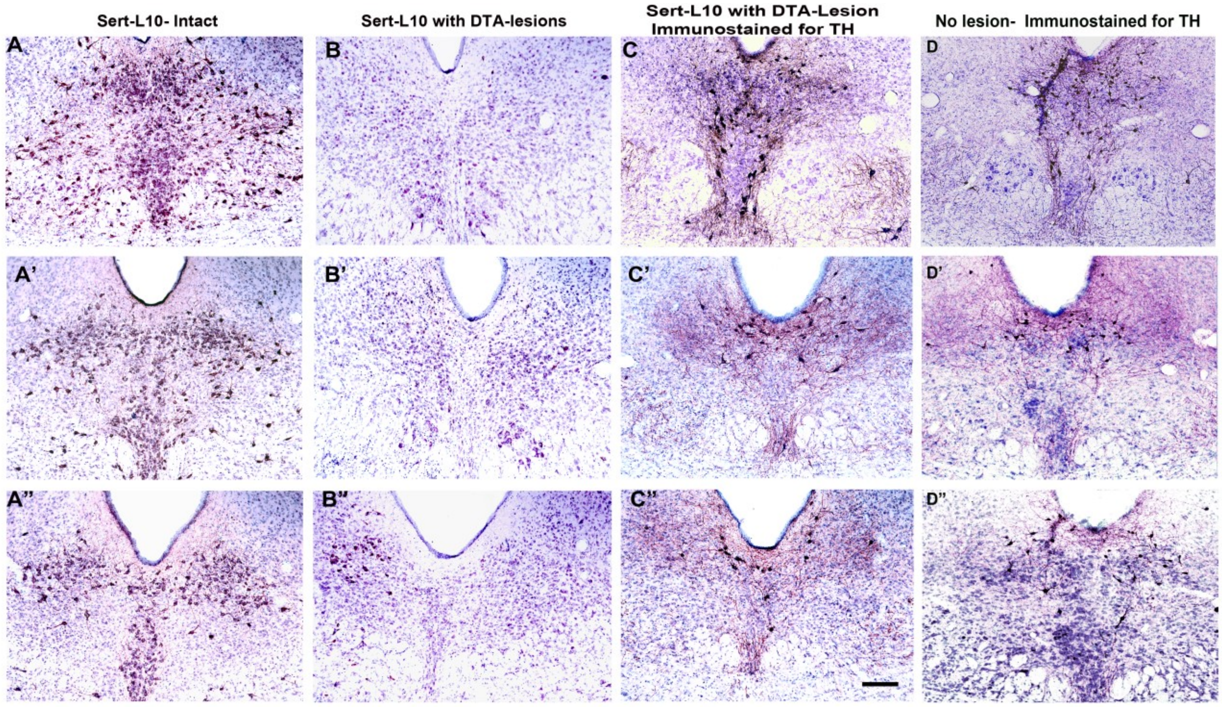


**Supplementary Figure 2** AAV-DTA induced selective killing of the serotonergic neurons in the DR of Sert-Cre mice: **A-C** shows sections through the DR from rostral to caudal levels in an intact Sert-L10 mouse (A-A") and one with DTA-induced deletion of $DR_{Sert}$ neurons (B-B"). The serotonin neurons in **A** and **B** are GFP positive, and are immunostained for GFP (brown), while background non-serotonergic neurons are shown with thionin staining. Panels **C-C"** show sections adjacent to those in panel B and in Fig. 1C lower panel, immunostained for tyrosine hydroxylase (TH, brown), and shows the presence of intact dopaminergic neurons in the area that was injected with AAV-DTA. Panels **D-D"** show the brain sections from the control un-lesioned mouse immune-stained for TH. The presence of intact Nissl's stained cells and dopaminergic cells demonstrates that the AAV-DTA injection selectively kills the serotonergic neurons. Scale= 100µm, seen in n=3 mice.

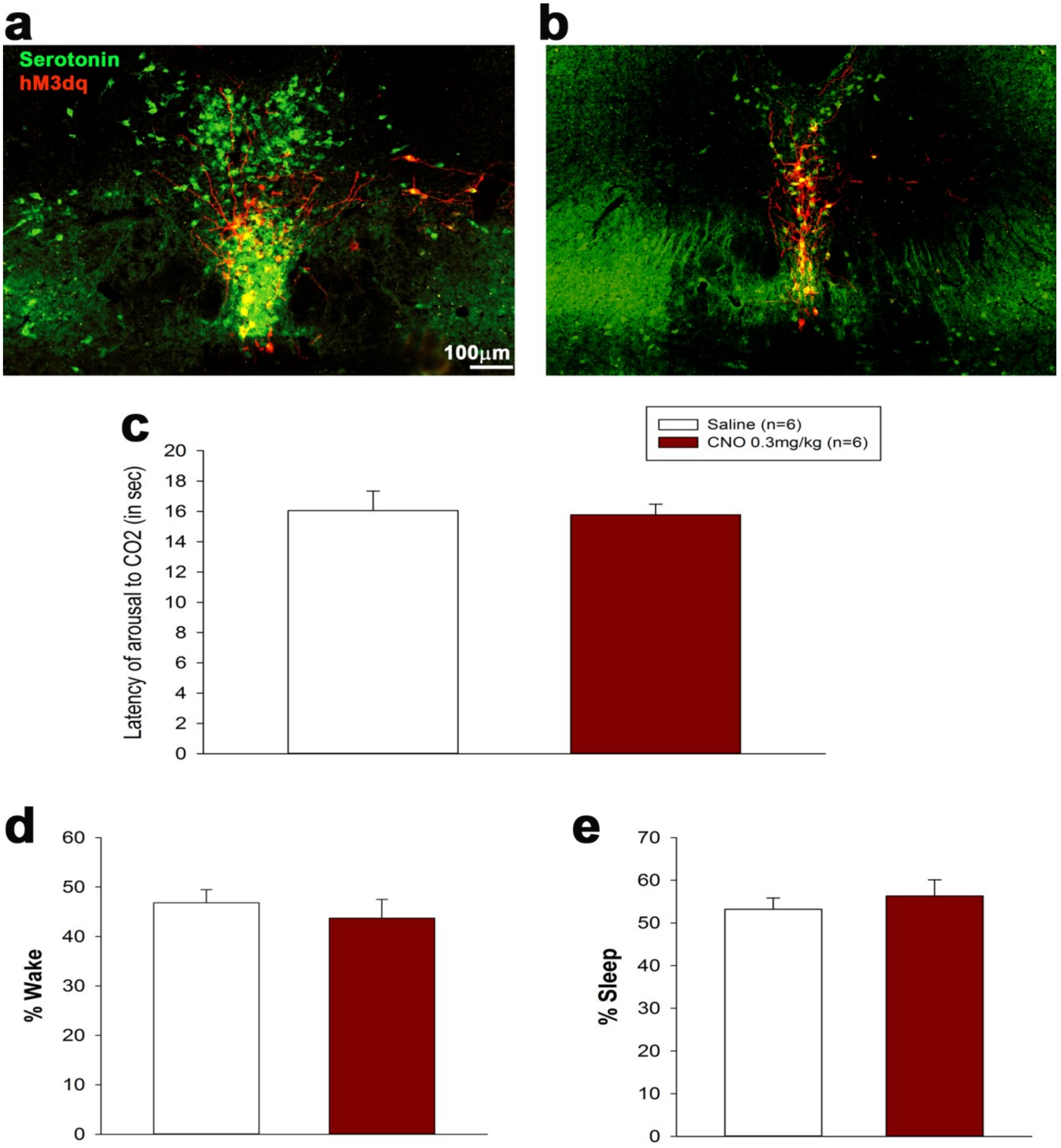


**Supplementary Figure 3** Chemogenetic activation of $DR_{Sert}$ neurons and its effect on the latency of arousal and sleep-wake: **a&b** are the photomicrographs of the brain sections stained for serotonin (green) and dsRed (red) of the mouse injected with AAV expressing hM3Dq in the dorsal raphe (DR), (seen in n=6 mice). Upon intraperitoneal injection of the CNO (0.3mg/kg), these mice (n=6) showed similar latency of arousal to the 10% $CO_2$ as the ones that received saline injection (**c**). Also, activation of $DR_{Sert}$, while these mice are recorded in plethysmograph, also spent similar amount of time in wake and sleep after injection of CNO than compared with saline (**d and e**). Data in c-e are presented as mean values ± SEM.

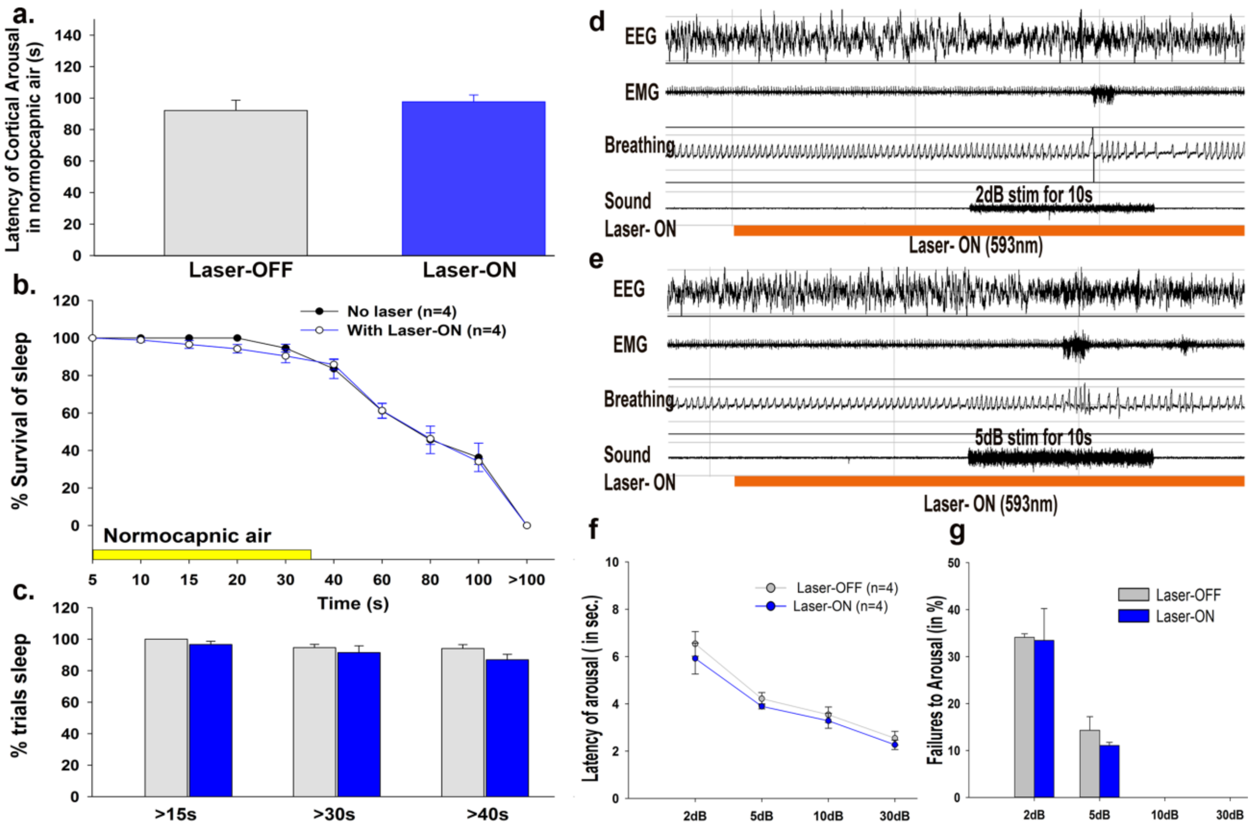


**Supplementary Figure 4** EEG arousals in response to normocapnic air and acoustic stimulus. a-c, Graphs showing the latency to arousal (mean ± SEM) during the Laser-ON and Laser-OFF conditions (n=4) in the presence of the normocapnic gas instead of CO2. The survival curves during the normocapnia air (b) and the arousal at the 15, 30 and 45s were comparable during laser ON and Laser OFF with normocapnic air (c). d,e, Representative figures showing normal arousals to the 2dB- 30dB, 4 kHz acoustic stimulus with Laser-ON and compared with laser-OFF (n=4). f, Latencies of cortical arousal (in sec; mean ± SEM) are compared at different sound intensities (x-axis) in the $DR^{Sert}$ mice injected with ArchT, which showed no significant difference in the latency to wake up to the sound, without laser (black) and with 60s Laser-ON (blue), in animals in which CO2 arousal was suppressed in the Laser-ON condition. G, shows the percentage of trials which show arousal in response to different sound intensities (x-axis) in the $DR^{Sert}$ mice in both laser-ON and laser-OFF conditions. Data in a-c and f, g are presented as mean values ± SEM.

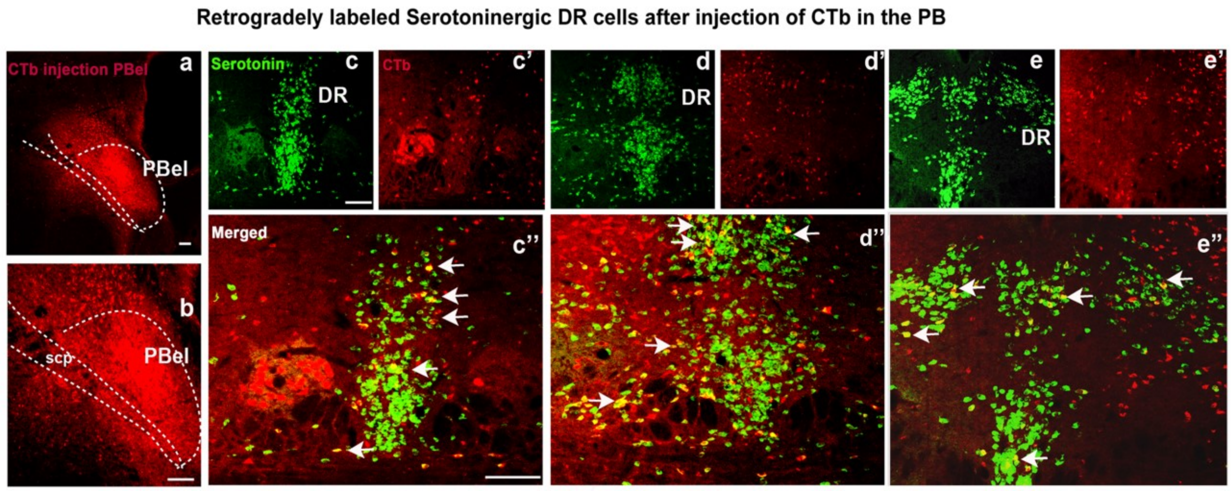


**Supplementary Figure 5** DR$^{Sert}$ input to PB: shows the confirmation of the DR$^{sert}$ to PB input by retrograde labeling in DR$^{Sert}$ cells, after injection of CTb in PB (For a-e, n=4). Panel c, d, and e show three rostral to caudal levels through the DR. Panels c, d, and 3 show serotonin neurons in green; c', d' and e' show CTb retrograde labeling in red; and c", d", and e" show merged images at higher magnification. Scale bars in a-e" = 100 µm.
