# Supplementary material for: Role of serotonergic dorsal raphe neurons in hypercapnia-induced arousals
Source: Nat Commun. 2020 Jun 2;11:2769. doi: 10.1038/s41467-020-16518-9 (PMC7265411; doi:10.1038/s41467-020-16518-9)
Supplement: Supplementary file 2 — Reporting Summary [file 41467_2020_16518_MOESM2_ESM.pdf]

nature research

Corresponding author(s): Clifford B. Saper

Last updated by author(s): 03/04/2020

Reporting Summary

Nature Research wishes to improve the reproducibility of the work that we publish. This form provides structure for consistency and transparency in reporting. For further information on Nature Research policies, see Authors & Referees and the Editorial Policy Checklist .

Statistics

For all statistical analyses, confirm that the following items are present in the figure legend, table legend, main text, or Methods section.

n/a	Confirmed	
☐	☒	The exact sample size (*n*) for each experimental group/condition, given as a discrete number and unit of measurement
☐	☒	A statement on whether measurements were taken from distinct samples or whether the same sample was measured repeatedly
☐	☒	The statistical test(s) used AND whether they are one- or two-sided<br>*Only common tests should be described solely by name; describe more complex techniques in the Methods section.*
☐	☒	A description of all covariates tested
☐	☒	A description of any assumptions or corrections, such as tests of normality and adjustment for multiple comparisons
☐	☒	A full description of the statistical parameters including central tendency (e.g. means) or other basic estimates (e.g. regression coefficient) AND variation (e.g. standard deviation) or associated estimates of uncertainty (e.g. confidence intervals)
☐	☒	For null hypothesis testing, the test statistic (e.g. *F*, *t*, *r*) with confidence intervals, effect sizes, degrees of freedom and *P* value noted<br>*Give P values as exact values whenever suitable.*
☒	☐	For Bayesian analysis, information on the choice of priors and Markov chain Monte Carlo settings
☐	☒	For hierarchical and complex designs, identification of the appropriate level for tests and full reporting of outcomes
☐	☒	Estimates of effect sizes (e.g. Cohen's *d*, Pearson's *r*), indicating how they were calculated

Our web collection on statistics for biologists contains articles on many of the points above.

Software and code

Policy information about availability of computer code

Data collection	EEG, EMG, respiration, and CO2 and O2 levels were fed into an Axon Digidata 1322A analog-to-digital converter and the signals were acquired using Axoscope software (Molecular Devices, Foster City, CA, USA).<br>Additional Softwares used in data collection:<br>1. Axoscope v.10 (Molecular Devices, Foster City, CA)<br>2. Clampfit v.10- a module of Axoscope v.10<br>3. Sirenia v. 2.10 (Pinnacle Technology Inc.)
Data analysis	Softwares for Data Analysis:<br>1. Sleep Sign software R. 3.3 (Kissie Comtec Co. Ltd., Matsumoto, Nagano, Japan)<br>2. Clampfit v. 10- a module of Axoscope v.10.<br>3 Sigmal Plot v.12.3 (Systat Software Inc.)

For manuscripts utilizing custom algorithms or software that are central to the research but not yet described in published literature, software must be made available to editors/reviewers. We strongly encourage code deposition in a community repository (e.g. GitHub). See the Nature Research guidelines for submitting code & software for further information.

Data

Policy information about availability of data

All manuscripts must include a data availability statement. This statement should provide the following information, where applicable:

- Accession codes, unique identifiers, or web links for publicly available datasets
- A list of figures that have associated raw data
- A description of any restrictions on data availability

All data generated to support the findings of this study are available from the corresponding author upon reasonable request.

Field-specific reporting

Please select the one below that is the best fit for your research. If you are not sure, read the appropriate sections before making your selection.

- [x] Life sciences
- [ ] Behavioural & social sciences
- [ ] Ecological, evolutionary & environmental sciences

For a reference copy of the document with all sections, see nature.com/documents/nr-reporting-summary-flat.pdf

Life sciences study design

All studies must disclose on these points even when the disclosure is negative.

Sample size	Using SigmaPlot 12.3, we tested the sample size and power of the tests post hoc and found that the power of each statistical test was at least 80% at alpha= 0.05, suggesting adequate sample sizes for all the experiments. We also chose the sample sizes based on our own published work on similar lines (Kaur et al., 2013 and 2017) and conform with others in the field (Buchanan et al., 2015, Smith et al., 2018) that worked on the role of serotonin in regulating waking up to CO2.
Data exclusions	None
Replication	Replication: We have included the reproducibility statement in all the figure legends that show micrographs. Data was reproduced in at least n=3 mice, before reporting it as a representative micrograph.
Randomization	Randomization: As mentioned in the manuscript, Each of these protocols were repeated for each mouse for two days; on one of the days the laser was switched on (Laser-ON) and on the other day the laser was off (Laser-OFF), again in random order. On the Laser-ON protocol, a 593nm laser was ON for 60s followed by 5 mins off, and this was repeated 20 times per session. In the Laser-OFF condition, everything was the same, except that the laser light was not turned on. Twenty seconds after the scheduled onset of the laser, the gas intake for the plethysmograph was switched either to normocapnic air (21% O2, 79% N2) or hypercapnic air (10% CO2, 21% O2, and 69% N2) for 30 sec.
Blinding	Blinding: As mentioned in the manuscript, The software autoscored each epoch using an algorithm that identified three behavioral states based on EEG and EMG. The autoscored data were then checked at least twice visually for movement and any other artifact and to correct automatic state classification by an unbiased scorer blind to the treatment groups (MAK and RCT). Similar blinding of the treatment groups was also used for the neuronal counting data done by the unbiased scorers.

Reporting for specific materials, systems and methods

We require information from authors about some types of materials, experimental systems and methods used in many studies. Here, indicate whether each material, system or method listed is relevant to your study. If you are not sure if a list item applies to your research, read the appropriate section before selecting a response.

Materials & experimental systems

n/a	Involved in the study	
[ ]	[x]	Antibodies
[x]	[ ]	Eukaryotic cell lines
[x]	[ ]	Palaeontology
[ ]	[x]	Animals and other organisms
[ ]	[ ]	Human research participants
[x]	[ ]	Clinical data

Methods

n/a	Involved in the study	
[x]	[ ]	ChIP-seq
[x]	[ ]	Flow cytometry
[x]	[ ]	MRI-based neuroimaging

Antibodies

Antibodies used	1. GFP - Rabbit anti-GFP, 1:10K, ThermoFisher Scientific, Cat- A11122, RRID:AB_10073917<br>2. serotonin antibody - Rabbit anti-Serotonin, ImmunoStar, 1:5K, Cat- 20080, RRID: AB_572263.<br>3. Cholera Toxin b (reterograde tracer)- anti CTb- 1:30K, Cat# 703, RRID:AB_10013220, List Biological Laboratories Inc., CA.<br><br>Additional antibodies:<br>Secondary antibodies - AF-488 (Catalog #- A32790, RRID- AB_2762833) and AF-Cy3 (Catalog#- A-10521 and RRID_ AB_2534030) were used in 1:200 dilution (ThermoFischer Scientific, USA)
Validation	Validation:<br>1. Rabbit anti GFP- Immunogen: The GFP was isolated directly from the jellyfish Aequorea victoria. It did not show immunostaining when primary antibody was omitted and no staining in the mouse brain tissue that was not injected with viral vection expressing GFP.<br>2. Rabbit anti Serotonin- Immunogen: Serotonin. The antiserum demonstrates significant labeling of rat hypothalamus, raphe

nuclei and spinal cord using indirect immunofluorescent and biotin/avidin-HRP techniques. Staining is completely eliminated by pretreatment of the diluted antibody with 25 micro g of serotonin/BSA. Cross reactivity of Serotonin antisera was examined. With 5micro g, 10 micro g and 25 micro g amounts the following substances did not react with diluted Serotonin antisera using the Bn-SA/HRP labeling method: 5-hydroxytryptophan, 5-hydroxyindole -3- aceticacid, and dopamine.

3. Goat anti Cholera toxin b- Immunogen- Cholera toxin subunit b. The antibody show no reaction in the mouse brain not injected the cholera toxin subunit b

Animals and other organisms

Policy information about studies involving animals; ARRIVE guidelines recommended for reporting animal research

Laboratory animals

Mice used:
1. Slc6a4 transgenic BAC-Cre recombinase driver line (Sert-Cre), males, of 8-26 weeks)
2.R26-lox-STOPlox-L10-GFP “Cre-reporter”, used all males, of 8-26 weeks
3. CGRPCreER mice, males, 8-26 weeks
Housing conditions: Animals were maintained on a 12 h light/dark cycle with ad libitum access to water and food and were singly housed after surgery. Male litter-mates were randomly assigned to the experimental groups. All animal procedures met National Institutes of Health standards, as described in the Guide for the Care and Use of Laboratory Animals, and all protocols were approved by the Beth Israel Deaconess Medical Center Institutional Animal Care and Use Committee.
Temperature and humidity data for the animal housing is now included in the manuscript.

Wild animals

None

Field-collected samples

None

Ethics oversight

All protocols were approved by the Beth Israel Deaconess Medical Center Institutional Animal Care and Use Committee

Note that full information on the approval of the study protocol must also be provided in the manuscript.

Human research participants

Policy information about studies involving human research participants

Population characteristics

NA

Recruitment

NA

Ethics oversight

NA

Note that full information on the approval of the study protocol must also be provided in the manuscript.
